# Tissue-resident memory CD8^+^ T cells amplify anti-tumor immunity by triggering antigen spreading through dendritic cells

**DOI:** 10.1038/s41467-019-12319-x

**Published:** 2019-09-27

**Authors:** Evelyn Menares, Felipe Gálvez-Cancino, Pablo Cáceres-Morgado, Ehsan Ghorani, Ernesto López, Ximena Díaz, Juan Saavedra-Almarza, Diego A. Figueroa, Eduardo Roa, Sergio A. Quezada, Alvaro Lladser

**Affiliations:** 1 Laboratory of Immunoncology, Fundación Ciencia & Vida, Santiago, Chile; 2 Cancer Immunology Unit, Research Department of Haematology, University College London Cancer Institute, London, UK; 3 Facultad de Medicina y Ciencia, Universidad San Sebastián, Santiago, Chile

**Keywords:** Melanoma, Tumour immunology, Immunological memory, Immunization, Dendritic cells

## Abstract

Tissue-resident memory CD8^+^ T (Trm) cells mediate potent local innate and adaptive immune responses and play a central role against solid tumors. However, whether Trm cells cross-talk with dendritic cells (DCs) to support anti-tumor immunity remains unclear. Here we show that antigen-specific activation of skin Trm cells leads to maturation and migration to draining lymph nodes of cross-presenting dermal DCs. Tumor rejection mediated by Trm cells triggers the spread of cytotoxic CD8^+^ T cell responses against tumor-derived neo- and self-antigens via dermal DCs. These responses suppress the growth of intradermal tumors and disseminated melanoma lacking the Trm cell-targeted epitope. Moreover, analysis of RNA sequencing data from human melanoma tumors reveals that enrichment of a Trm cell gene signature associates with DC activation and improved survival. This work unveils the ability of Trm cells to amplify the breath of cytotoxic CD8^+^ T cell responses through DCs, thereby strengthening anti-tumor immunity.

## Abstract (web summary)

Immunotherapy can induce antigen spreading of antitumor T cell response, which correlates with better outcomes. Here the authors show that tissue-resident memory CD8 T cells promote antigen spreading via lysing tumor cells and promoting their uptake and cross-presentation by dendritic cells, thereby eliciting de novo T cell responses.

## Introduction

Cytotoxic CD8^+^ T lymphocytes (CTL) play a pivotal role in providing effective antigen-specific immunity against tumors. Tumor-specific CTL responses are initiated at secondary lymphoid organs when naïve CD8^+^ T cells are activated by mature migratory dendritic cells (DCs) presenting tumor-derived antigens on MHC class I molecules^1,2^. Antigen-specific CD8^+^ T cells massively proliferate and differentiate into cytotoxic effector T (Teff) cells, which can then migrate to peripheral tissues and recognize cancer cells through their T-cell receptor (TCR). CTL destroy target tumor cells through mechanisms including release of granules containing perforin and granzymes and inducing FasL-mediated apoptosis. To achieve long-lasting anti-tumor immunity, it is necessary to establish memory CD8^+^ T-cell responses^3,4^. Classically, the circulating memory compartment consists of central-memory (Tcm) and effector-memory (Tem) CD8^+^ T cells^5^. Tcm cells circulate between secondary lymphoid organs and blood, whereas Tem cells circulate between blood and non-lymphoid tissues^5^. In contrast to these circulating subsets, tissue-resident memory CD8^+^ T (Trm) cells stably reside in lymphoid and non-lymphoid tissues where they provide potent local innate and adaptive immune responses^6^. The remarkable ability of Trm cells to mediate protective immunity has prompted the development of more potent vaccination strategies by eliciting Trm cells against infectious diseases^7,8^. Evidence supporting a central role of Trm cells in tumor immunosurveillance has recently emerged from animal models^9,10^. We and others have demonstrated that antigen-specific Trm cell responses mediate strong tissue-restricted immunity against cutaneous melanoma and other tumor models^9,11–13^. However, the precise mechanisms by which Trm cells mediate enhanced anti-tumor immunity are poorly understood.

In human cancers, infiltration of CD103^+^ CD8^+^ T cells in solid tumors has been associated with longer survival in patients with breast, lung, endometrial, ovarian, cervical, urothelial and melanoma tumors^14–23^. Moreover, tumor-infiltration of Trm cells was recently associated with improved survival in melanoma patients that received an antibody blocking the inhibitory receptor PD-1^23^. T-cell residency across different tissues, including tumors, is defined by a distinctive gene expression program commanded by transcription factors, including Runx3, Blimp1, Hobit and Nur77^24–26^. Tissue adaptation involves constitutive upregulation of CD69, CD49a and CD103, which sustain enhanced ability of Trm cells to become established in the tumor niche and better suited to fight tumors. CD69 is a C-type lectin expressed by Trm cells from most tissues that render these cells unresponsive to tissue egress signals such as sphingosine-1-phosphate (S1P) by reducing the levels of S1P receptor^27^. CD49a, or α1β1 integrin, is an adhesion molecule that binds to the extracellular matrix proteins collagen and laminin^28^ and distinguish Trm cells with higher cytotoxic potential in skin and melanoma tumors^29,30^. Trm cells confined to epithelial barriers also express CD103 (integrin αEβ7), which binds to E-cadherin and facilitates their interaction with epithelial cells^31^. In CD8^+^ T cells isolated from lung tumors, CD103 molecules have been shown to distribute preferentially near the immune synapse formed with the target tumor cell and to facilitate cytotoxic vesicle degranulation in an E-cadherin-dependent fashion^32^. Thus, tissue adaptation-related features and enhanced cytotoxicity may contribute to the superior protective potential of Trm cells in human solid cancers.

Complementary to their cytotoxic activity, Trm cells secrete large amounts of effector molecules, most prominently IFN-γ and TNF-α, which can activate other immune cells with anti-tumor potential. In the context of viral infections, IFN-γ produced by Trm cells triggers an innate-like alarm state characterized by the production of chemokines and antimicrobial molecules in the tissue and the recruitment of circulating memory CD8^+^ T cells^33,34^. In cancer, the presence of a tissue-resident gene signature is associated with higher density and enhanced cytotoxicity of CTLs infiltrating lung and breast tumors^16,35^. In viral models, Trm cell-derived IL-2 has been shown to promote upregulation of granzyme B in NK cells^36^. Additionally, antigen-specific activation of Trm cells leads to the production of TNF-α, which promotes rapid DC maturation and up-regulation of the lymph node homing chemokine receptor CCR7^36,37^. However, whether Trm cell activation in the tissue causes DC migration to draining lymph nodes and the subsequent initiation of protective CTL immune responses remains unknown. Particularly in the context of the tumor microenvironment, the innate–like capabilities of Trm cells and their potential cross-talk with DCs remain largely unexplored. We hypothesized that Trm cells cooperate with DCs to support anti-tumor immunity by initiating secondary T-cell responses against tumor-derived antigens.

Here, we demonstrate that skin Trm cell activation promotes maturation and trafficking to draining lymph nodes of migratory dermal DCs. Trm cell-mediated melanoma rejection leads to the spreading of circulating CTL responses against tumor-derived neo- and self-antigens that protects against intradermal tumors and disseminated melanoma lacking the Trm cell-targeted antigen. Transcriptional analysis of human melanoma tumors reveals that a Trm cell gene signature associates with DC activation and improved survival. This work highlights the ability of Trm cells to cross-talk with DCs and orchestrate the broadening of anti-tumor CTL immunity.

## Results

### Trm cells trigger maturation and migration of dermal DCs

**Fig. 1:**
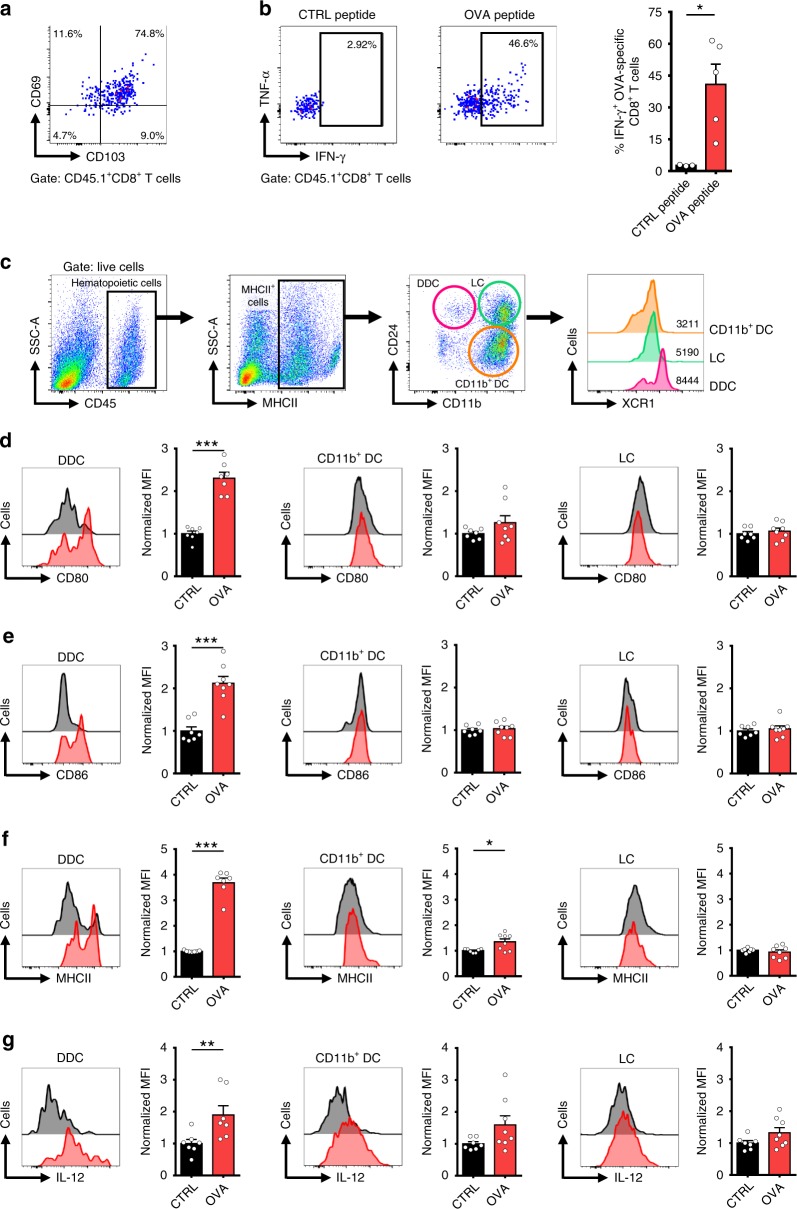
Skin Trm cell activation induces maturation of dermal DCs. C57BL/6 mice bearing OVA-specific skin Trm cells and depleted of circulating CD8^+^ T cells by administration of an anti-CD8 antibody were intradermally inoculated with control (CTRL) or OVA_(257-264)_ (OVA) peptides to activate Trm cells. **a**, **b** OVA-specific CD45.1^+^ CD8^+^ T cells in the skin were analyzed 6 h later by intracellular cytokine staining and flow cytometry. **a** Representative pseudocolor dot-plot showing CD69 and CD103 expression. **b** Representative pseudocolor dot-plots and graph showing IFN-γ and TNF-α production by skin Trm cells. **c**–**g** DCs in the skin were analyzed 24 h after peptide stimulation. **c** Gating strategy used to identify skin DC subpopulations, including CD11b^+^ DCs, Langerhans cells (LC) and dermal DCs (DDC) and representative histograms showing XCR1 expression in each subset. **d**–**g** Representative histograms (black: CTRL; red: OVA) and graphs showing of the expression of CD80, CD86, MHCII and IL-12 of each skin DC subset. For quantification, the geometric mean fluorescence intensity (MFI) was normalized relative to the average of the control group. Pooled data from two independent experiments, *n* = 7 mice in the group treated with control peptide and n = 8 mice in the group stimulated with OVA_(257-264)_ peptide. Bars are the mean ± SEM. *p *<* 0.05, **p < 0.01, ***p < 0.001 by Mann-Whitney unpaired test

**Fig. 2:**
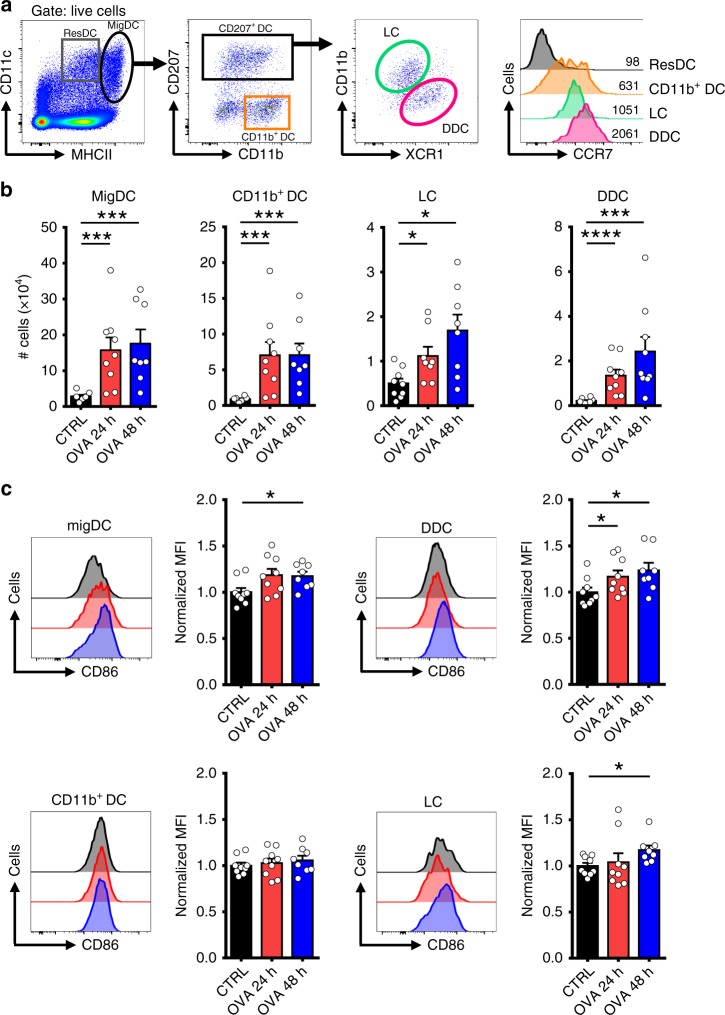
Trm cell activation promotes migration of dermal DCs. C57BL/6 mice bearing OVA-specific skin Trm cells and depleted of circulating CD8^+^ T cells by administration of an anti-CD8 antibody were intradermally inoculated with control (CTRL) or OVA_(257-264)_ peptides followed by analysis of inguinal draining lymph nodes after 24 h (OVA 24 h) and 48 h (OVA 48 h). **a** Gating strategy used to identify the different DC subsets in skin draining lymph nodes, including: lymph node-resident DCs (ResDC), migratory DCs (MigDC), CD11b^+^ DCs, LCs and DDCs. Representative histograms showing CCR7 expression on relevant subpopulations. **b** Quantification of total numbers of the different DC subsets. **c** Representative histograms (black: CTRL; red: OVA 24 h; blue: OVA 48 h) and graphs showing CD86 expression of each DC population. For quantification, the geometric mean fluorescence intensity (MFI) was normalized relative to the average of the control group. Pooled data from two independent experiments *n* = 9 mice per group. Bars are the mean ± SEM. *p *<* 0.05, **p < 0.01, ***p < 0.01 by Mann-Whitney unpaired test

We first aimed to study the potential interplay between Trm cells and migratory DCs in the skin. To this end, we generated ovalbumin (OVA)-specific skin Trm cells in mice using intradermal (i.d.) vaccination. This was followed by administration of an anti-CD8 antibody during the memory phase of the response (>4 weeks post vaccination), which efficiently deplete circulating CD8^+^ T cells (Supplementary Fig. 1a, b), including circulating memory and effector OVA-specific CD8^+^ T cells in lymphoid and non-lymphoid tissues, as previously shown^11^. Then, depletion-resistant Trm cells (Fig. 1a) were specifically activated by i.d. injection of the immunodominant OVA_(257-264)_ peptide, readily producing IFN-γ and TNF-α within the first 6 h (Fig. 1a, b). Interestingly, we observed that skin XCR1^+^ conventional type 1 DCs (cDC1), also known as dermal DCs or DDCs (Fig. 1c) upregulated CD80, CD86, MHC class II and IL-12 molecules 24 h after Trm cell activation (Fig. 1d–g). These data indicate that Trm cells induce maturation of dermal DCs, which are specialized in antigen cross-presentation and priming of CD8^+^ T cells^38^. Then, we analyzed the presence of skin migratory DCs in draining lymph nodes based on the expression of high levels of MHC class II, CD207 (langerin) and CCR7 (Fig. 2a). We observed a marked accumulation of different migratory DC subsets at 24 and 48 h after Trm cell activation, including dermal DCs, Langerhans cells (LCs) and CD11b^+^ DCs (Fig. 2b). Among these subsets, dermal DCs displayed upregulated expression of the maturation marker CD86 (Fig. 2c). These results indicate that antigen-specific activation of Trm cells triggers maturation and migration to draining lymph nodes of skin-derived dermal DCs, revealing a cross-talk among these cells.

### Trm cells spread CTL responses against tumor neo-antigens

**Fig. 3:**
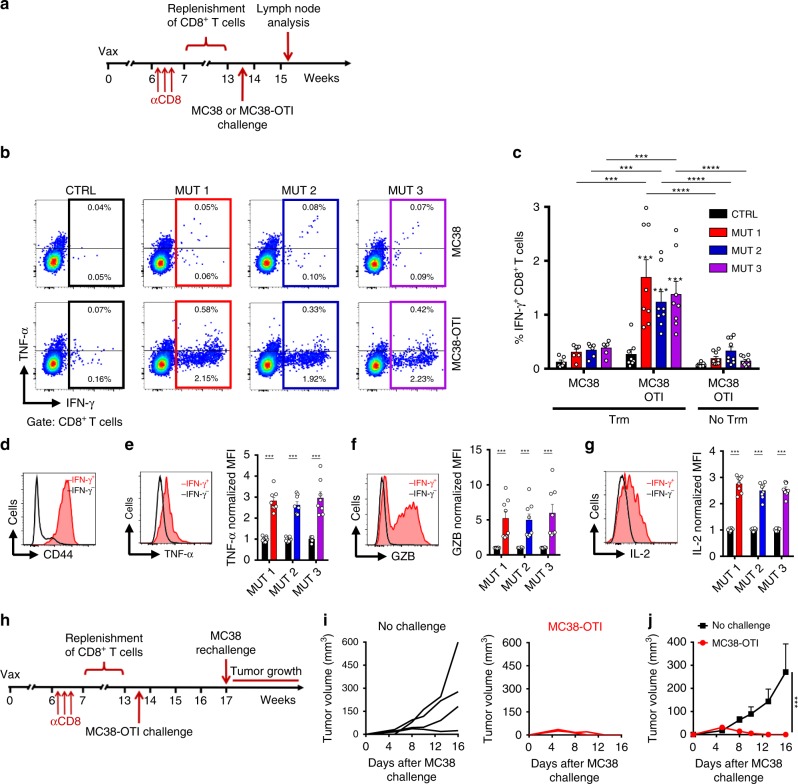
Trm cell-mediated tumor rejection spread CTL responses against neo-antigens. **a**–**g** C57BL/6 mice bearing OVA-specific skin Trm cells were depleted of circulating CD8^+^ T cells by administration of an anti-CD8 antibody. Eight weeks later, mice were challenged intradermally with MC38 (*n* = 6) or MC38-OTI (*n* = 8) cells. A group of unvaccinated mice (No Trm) was challenged with MC38-OTI cells (*n* = 9) as additional control. **b**–**g** Expression of IFN-γ, CD44, TNF-α, granzyme B and IL-2 was analyzed 12 days after tumor challenge in CD8^+^ T cells present in draining lymph nodes after ex vivo stimulation with control (CTRL) or neo-epitope peptides (MUT 1, MUT 2 and MUT 3) followed by intracellular cytokine staining. **a** Experimental timeline scheme. **b** Representative pseudocolor dot-plots showing IFN-γ and TNF-α production from a mouse challenged with MC38 (upper panels) and MC38-OTI (lower panels). **c** Graph showing the frequencies of IFN-γ-producing CD8^+^ T cells. **d**–**g** Representative histograms (black histograms: IFN-γ^-^ CD8^+^ T cells; red histograms: IFN-γ^+^ CD8^+^ T cells) and graphs (black bars: IFN-γ^-^ CD8^+^ T cells; colored bars: IFN-γ^+^ CD8^+^ T cells) showing the expression of CD44 (**d**), TNF-α (**e**), granzyme B (**f**), and IL-2 (**g**). For quantification, the geometric mean fluorescence intensity (MFI) was normalized relative to the average of IFN-γ^-^ CD8^+^ T cells. **h**-**j** Mice bearing OVA-specific Trm cells that rejected MC38-OTI cells, were re-challenged with MC38 cells (MC38-OTI, *n* = 4) and tumor growth was monitored. Mice that did not received initial challenge (No challenge, *n* = 4) were used as controls. **h** Experimental timeline scheme. **i** Individual curves showing tumor growth. **j** Graphs showing the mean of tumor volume. Bars are the mean ± SEM. ***p ≤ 0.001, ****p ≤ 0.0001 by Mann-Whitney unpaired test for **c**, **e**–**g** and two-way ANOVA Bonferroni post-hoc test for **j**

Given the ability of Trm cells to activate migratory skin DCs and also mediate tumor-cell killing^11,36^, we hypothesized that antigen-specific Trm cell-mediated tumor rejection would lead to the generation of secondary responses against other tumor-derived antigens, a phenomenon known as antigen spreading. To test this, mice bearing OVA-specific Trm cells were depleted from circulating CD8^+^ T cells and let during 8 weeks to replenish this compartment (Supplementary Fig. 1c). This allows resetting the endogenous repertoire in terms of specificity while maintaining OVA-specific Trm cells in the skin. Then, mice were challenged i.d. with MC38 cells expressing the OVA_(257-264)_ peptide (MC38-OTI), which were rejected by OVA-specific Trm cells. As controls, unvaccinated mice (no Trm) challenged with MC38-OTI and Trm cell-bearing mice challenged with parental MC38 were used. Secondary CD8^+^ T-cell responses raised against highly relevant neo-epitopes present in MC38 cell line^39^ were analyzed 12 days later in tumor-draining lymph nodes (Fig. 3a). To this end, lymph node cells were ex vivo stimulated with neo-epitopes carrying missense mutations MUT 1 (SIIVFNLL from *Dpagt1* gene), MUT 2 (AQLANDVVL from *Reps1* gene) and MUT 3 (ASMTNMELM from *Adpgk* gene) and the production of effector molecules was analyzed by intracellular staining and flow cytometry. In contrast to control groups, rejection of MC38-OTI mediated by OVA-specific Trm cells resulted in the expansion of CD8^+^ T cells specific to all neo-epitopes tested (Fig. 3b, c), detected as IFN-γ-producing CD8^+^ T cells, which also expressed high levels of CD44 (Fig. 3d). These results indicate that spreading of CD8^+^ T-cell responses to multiple antigens is triggered by Trm cell-mediated tumor rejection. Neo-epitope-specific CD8^+^ T cells displayed high expression of other effector molecules, such as TNF-α, granzyme B and IL-2 (Fig. 3e, g), which is consistent with anti-tumor cytotoxic activity. Indeed, these mice were able to reject a re-challenge with MC38 cells, which express the neo-epitopes but cannot be recognized by OVA-specific Trm cells (Fig. 3h-k).

### Trm cells promote melanoma-antigen spreading through DCs

**Fig. 4:**
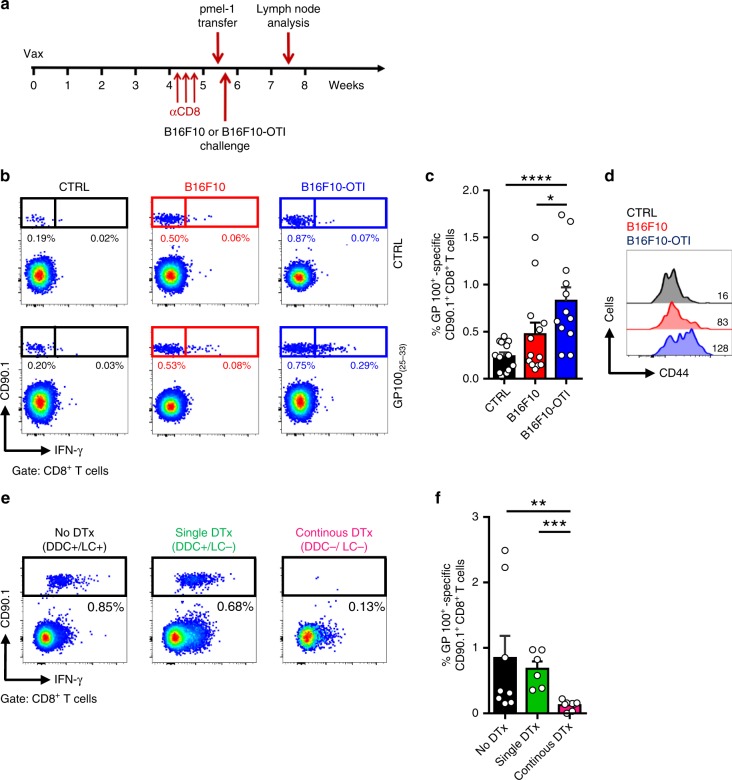
Trm cells promote melanoma-antigen spreading through dermal DCs. C57BL/6 and Langerin-DTR mice bearing OVA-specific skin Trm cells and depleted of circulating CD8^+^ T cells were intravenously transferred with gp100-specific CD90.1^+^ pmel-1 CD8^+^ T cells and one day later challenged intradermally with B16F10-OTI melanoma cells. Control groups were either unchallenged (CTRL) or challenged with or B16F10 melanoma cells that do not activate Trm cells. Analysis of gp100-specific pmel-1 CD8^+^ T cells was performed 12 days after tumor challenge in inguinal draining lymph nodes stimulated ex vivo with cognate gp100_(25-33)_ peptide to analyze IFN-γ production by intracellular cytokine staining. **a** Experimental scheme. **b** Representative pseudocolor dot-plots showing the frequency of gp100-specific pmel-1 CD8^+^ T cells and IFN-γ production. **c** Graph showing frequencies of gp100-specific pmel-1 CD8^+^ T cells. **d** Representative histograms (CTRL: black histogram, upper panel; B16F10: red histogram, middle panel; B16F10-OTI: blue histogram, lower panel) and geometric mean fluorescence intensity values of CD44 expression in CD90.1^+^ pmel-1 CD8^+^ T cells. **e**, **f** Representative pseudocolor dot-plots (**e**) and graph (**f**) showing the frequency of gp100-specific pmel-1 CD8^+^ T cells and IFN-γ production in Langerin-DTR mice that were either: non-depleted of LCs and DDCs (No DTx; DDC+/LC+ ); depleted of only LCs by administrating a single dose of diphtheria toxin (DTx) 14 days before the tumor challenge (Single DTx; DDC+/LC-); or depleted of both DDCs and LCs through the continuous administration of DTx (Continous DTx; DDC-/LC-). Pooled data from three independent experiments in **b**–**d**, *n* = 13 mice per group, and two independent experiments in **e**–**f**, *n* = 7-8 mice per group. Bars are the mean ± SEM. *p < 0.05, **p ˂0.01, ***p < 0.001, ****p < 0.0001, by Mann-Whitney unpaired test

To confirm these results in a relevant metastatic melanoma model, we used B16F10 cells, which are less immunogenic and express melanocyte-associated self-antigens, such as gp100. Favorably, responses against H-2 Kb-restricted gp100_(25-33)_ peptide can be tracked by transferring congenic TCR-transgenic CD8^+^ T cells from pmel-1 mice without the need to wait for the replenishment of the endogenous repertoire. Mice bearing OVA-specific Trm cells and devoid of circulating CD8^+^ T cells received i.v. transfer of pmel-1 CD8^+^ T cells (Fig. 4a). The following day, mice were challenged i.d. with B16F10 cells expressing the OVA_(257-264)_ peptide (B16F10-OTI), which are rejected by OVA-specific Trm cells, as previously shown by us^11^. After 12 days, the generation of gp100-specific CTL responses was analyzed in the draining lymph nodes. Control groups were either left unchallenged (CTRL) or challenged with B16F10 parental cell line that do not activate OVA-specific Trm cells. As compared to control groups, only mice challenged with B16F10-OTI presented a significant expansion of gp100-specific CD8^+^ T cells (Fig. 4b, c), which produced IFN-γ after ex vivo peptide stimulation and displayed high expression of CD44 (Fig. 4d), indicating that they were efficiently primed. Since both B16F10-OTI and B16F10 cells express gp100 but only B16F10-OTI can be recognized by OVA-specific Trm cells, these results suggest that melanoma recognition by Trm cells triggers the spreading of CD8^+^ T-cell responses to tumor-derived antigens.

To explore whether cross-presenting dermal DCs mediate antigen spreading, we carried out similar experiments using Langerin-DTR mice, which allow the selective depletion of CD207/langerin^+^ dermal DCs and LCs from the skin after diphtheria toxin (DTx) administration^40–42^. Taking advantage of the relatively faster repopulation of dermal DCs (~2 weeks) derived from bone marrow precursors, in comparison to LCs (>4 weeks) that arise from slowly proliferating skin precursors, we performed the B16F10-OTI challenge in mice devoid of only LCs (single DTx administration two weeks before challenge) or depleted of both LCs and dermal DCs (continuous DTx administration starting one day before challenge) (Supplementary Fig. 2)^43,44^. Similar to wild-type mice, pmel-1 CD8^+^ T cells were clonally expanded following B16F10-OTI challenge in Langerin-DTR mice that were not treated with DTx (No DTx; DDC+/LC+) or received a single DTx dose (Single DTx; DDC+/LC-) (Fig. 4e, f). Interestingly, the expansion of pmel-1 CD8^+^ T cells was severely reduced in the case of mice depleted of both dermal DCs and LCs by continuous DTx administration (Continuous DTx; DDC- LC-), indicating that dermal DCs are necessary for antigen spreading induced by Trm cells. If dermal DCs directly present tumor-derived antigens to naïve CD8 + T cells in the lymph nodes remain to be determined.

### Trm cell-induced CTL spreading suppresses melanoma growth

**Fig. 5:**
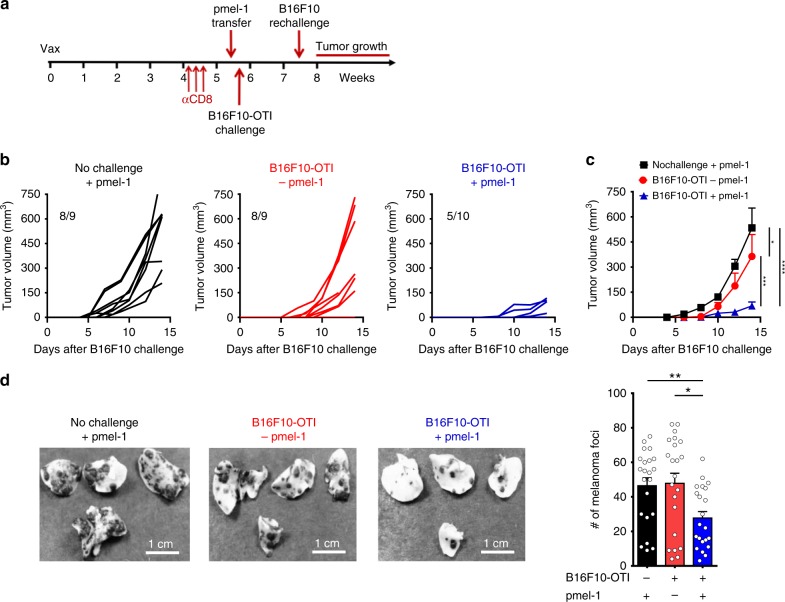
Trm cell-induced antigen spreading suppress melanoma growth. C57BL/6 mice bearing OVA-specific skin Trm cells and depleted of circulating CD8^+^ T cells were intravenously transferred with gp100-specific CD90.1^+^ pmel-1 CD8^+^ T cells and one day later challenged intradermally with B16F10-OTI cells (B16F10-OTI + pmel-1). Control groups were not challenged but received pmel-1 CD8^+^ T cells (no challenge + pmel-1) or challenged but did not received pmel-1 CD8^+^ T cells (B16F10-OTI - pmel-1). Two to three weeks later, mice were re-challenged intradermally in the contralateral flank (b-c) or intravenously (d) with B16F10 melanoma cells. **a** Experimental scheme. **b** Individual curves showing tumor growth. **c** Graph showing the mean of tumor volume in all groups **d** Representative picture of lungs and graph showing the number of melanoma foci. Pooled data from two independent experiments in **b**, **c** *n* = 10, and three independent experiments in **d** *n* = 22 mice. Bars are the mean ± SEM. *p ˂0.05, **p ˂0.01, ***p ≤ 0.001, and ****p ≤ 0.0001 by two-way ANOVA Bonferroni post-hoc test for **c**, and Mann-Whitney unpaired test for **d**

We next determined whether secondary CD8^+^ T-cell responses triggered by Trm cells were able to protect against B16F10 melanoma cells lacking OVA antigen and cannot be recognized by vaccination-induced Trm cells^11^. To this end, mice that rejected B16F10-OTI cells were injected 2–3 weeks later in the opposite flank with B16F10 melanoma cells (re-challenge) (Fig. 5a). Interestingly, these mice suppressed the growth of cutaneous tumors as compared to control mice that did not receive initial B16F10-OTI challenge (Fig. 5b,c), and therefore did not prime gp100-specific pmel-1 CD8^+^ T cells. In addition, no protection against B16F10 re-challenge was observed in mice that rejected B16F10-OTI melanoma but that did not receive transfer of pmel-1 CD8^+^ T cells (Fig. 5b, c), directly implicating the participation of primed pmel-1 CD8^+^ T cells in the anti-tumor effects observed. These results imply that Trm cell-mediated melanoma rejection triggers the spreading of CD8^+^ T-cell responses against melanoma-associated antigens, providing cross-protection against melanoma lacking Trm cell-targeted antigen. This can potentially be important to control highly heterogeneous tumors containing antigen-loss escape mutants.

To address the potential of Trm cell-induced gp100-specific CTL responses to protect against tumors disseminated in distant tissues, we repeated the previous experiment but substituted i.d. for i.v. B16F10 re-challenge to form disseminated pulmonary melanoma foci. Similar to i.d. re-challenge experiments, we observed that gp100-specific CTLs educated upon Trm cell-mediated melanoma rejection suppressed the formation of melanoma foci, as compared to unchallenged or non-transferred controls (Fig. 5d). These data suggest that Trm cells can orchestrate the generation of systemic CTL responses, which have the potential to protect against metastatic tumors.

### Trm cell-DC cross-talk in human melanoma

**Fig. 6:**
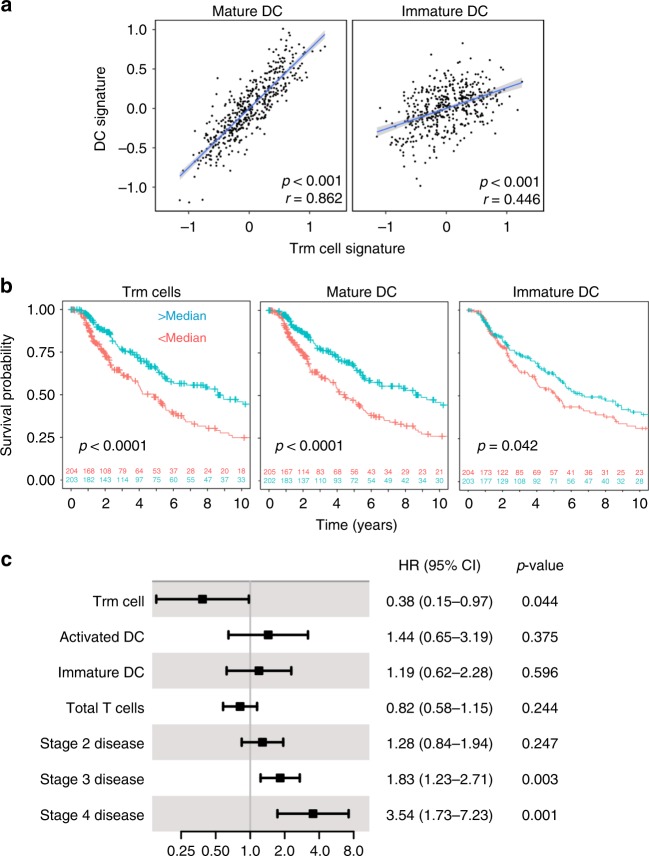
Trm cell signature correlates with DC maturation and improved survival. The relationship between previously described gene transcription signatures of Trm cells, DCs and survival was investigated in the TCGA melanoma cohort. **a** Correlation between Trm cell gene signature and either mature or immature DC gene signatures, summarized as enrichment z-scores (*n* = 468). Pearson correlation coefficients and associated p-values are shown. **b** Kaplan-Meier plots showing the overall survival of patients (*n* = 407 with available data) grouped according the median value of signatures (light blue curves: upper half; red curves: lower half) corresponding to Trm cells, mature DC and immature DCs. Log-rank p-values are shown. **c** Multivariable Cox survival regression carried out on the same cases represented in **b**. The forest plot shows the hazard ratio, 95% confidence interval and associated p-values for each variable in the model

Finally, we set out to determine whether there is evidence of cross-talk between Trm cells and DCs in human cancer. Using previously described gene signatures for Trm cells, activated and immature DCs^35,45^, we analyzed tumor transcriptomic data of patients with cutaneous melanoma available within The Cancer Genome Atlas^46,47^. We found a striking correlation between Trm cell and activated DC signatures (r = 0.862), with a weaker correlation between Trm cells and immature DCs (r = 0.446; Fig. 6a). This is in keeping with our finding that Trm cells promote DC maturation in mice and suggests a similar process may occur in human melanoma. Whilst both Trm and activated DC enrichment correlated with better overall survival, this association was weaker for immature DCs (Fig. 6b). As these signatures are correlated, we carried out multivariable Cox regression analysis to estimate their individual contributions to patient survival, additionally correcting for total T-cell infiltrate using a previously published signature^48^ and stage, showing Trm cell enrichment to remain a strong predictor of survival (Fig. 6c). These results suggest that that a similar Trm cell-DC cross-talk may occur in human melanoma.

## Discussion

There is compelling evidence supporting a central role for Trm cells in anti-tumor immunity. However, the precise mechanisms by which Trm cells can control solid tumors are just starting to be deciphered. Here we show that, in parallel to their well-documented direct tumor killing capability^49^, Trm cells can activate cross-presenting dermal DCs, resulting in the subsequent priming and expansion of new CD8^+^ T cells specific to tumor-derived neo- and self-antigens. Importantly, this secondary response confers protection against re-challenge with tumor cells lacking the antigen initially recognized by Trm cells. To our knowledge, this is the first study to show that Trm cells can orchestrate the broadening of circulating CTL responses via DCs to strengthen protective immunity. Interestingly, these secondary CTL responses were able to suppress the growth of cutaneous tumors and also disseminated melanoma in the lungs. The ability of Trm cells to trigger the generation of broader and systemic CTL responses to control disseminated tumors implies that they can overcome their tissue-restricted nature, spreading their protective potential to other organs. This notion is supported by our findings in human data indicating that a Trm cell gene signature positively correlates with overall survival.

This reverse flow of information from adaptive to innate immune responses was initially described during anti-viral immune responses. A few reports have demonstrated that following Trm cell activation, a strong innate-like alarm state is induced in the tissue through the production of a plethora of effector molecules^33^. Among these, TNF-α has been shown to promote maturation of DCs^36^. However, whether Trm cell-induced DC maturation results in the generation of new CD8^+^ T-cell responses had not previously been addressed. The present study reveals that such cross-talk between Trm cells and DCs occurs in the context of anti-tumor immunity and, more importantly, that it results in the propagation of circulating anti-tumor CTL responses.

The results obtained in mouse models are supported by human data showing a strong correlation between Trm cell and activated DC gene signatures in tumors from melanoma patients. The broader anti-tumor CTL responses triggered by Trm cells can eventually underlie the association between Trm cell infiltration and higher density of CTLs observed in some human solid tumors^16,35^, as well as the superior predictive potential and better response to immunotherapy that Trm cells have in comparison to total CD8^+^ T-cell infiltration^16^. This mechanism may have broader implications because Trm cell-infiltration has been shown to predict better clinical outcome in other types of solid tumors^14–23^. On the other hand, cross-presenting migratory DCs are key players in the generation of anti-tumor T-cell immunity and their absence abolishes the rejection of immunogenic tumors and decreases the response to immune checkpoint blockade and adoptive T-cell therapy^1,50–52^.

Emerging evidence indicates that effective anti-tumor immunity requires the coordinated action of tissue-resident and circulating T-cell compartments^12,53,54^. However, how these two compartments team-up to control tumors is poorly understood. It has been previously demonstrated that virus-specific Trm cells can recruit circulating bystander memory CD8^+^ T cells to the infection site after antigen recognition through the production of IFN-γ^33^. In tumor models, circulating Tcm cells have been shown to differentiate into Trm cells^12^. Here we show that Trm cells can increase the breadth of the circulating CD8^+^ T-cell repertoire. Our findings suggest a novel mechanism by which resident and circulating T cells can collaborate to fight tumors.

The mechanism described in this manuscript could be of particular relevance to control highly heterogeneous tumors, which represents a major challenge to oncological treatments and, in particular, immunotherapies. Indeed, tumor-cell exon sequencing has revealed that multiple regions inside the same tumor or different lesions in the same patient have divergent mutation patterns^55,56^ and probably a differential expression of antigens. Consequently, Trm cells derived from different metastasis of the same patient have a high interlesional TCR diversity^57^. On the other hand, the adaptive immune system exerts a selective pressure on tumor cells, driving the survival of more resistant cancer cell subpopulations, a phenomenon known as immune editing^58^. As a result, cancer cell clones that do not express immune targeted antigens can escape immune control and form new tumors^58^. In this regard, Trm cell responses can drive the control of resistant clones, such as antigen-loss escape mutants, by broadening anti-tumor CTL responses against multiple tumor-derived antigens, as shown here.

In summary, we propose that Trm cells represent a new orchestrator of anti-tumor immunity. Interestingly, it has been suggested that Trm cells are major targets of checkpoint blockade^57^ and that checkpoint blockade promotes Trm cell formation in tumors^12^. Hence, we envision that the ability of Trm cells to increase the breath of anti-tumor T-cell immunity via DCs may play an important role in cancer immunotherapy. Accordingly, recent studies have evidenced the importance of the cross-talk between DCs and CD8^+^ T cells for effective cancer immunotherapy^59^. Moreover, a recent study has revealed that PD-1 blockade leads to the expansion of new tumor-reactive T-cell clones in patients with advanced skin cancer^60^. In consequence, the development of therapeutic approaches, such as vaccines, T-cell-based therapies and monoclonal antibodies, that boost the ability of Trm cells to broaden anti-tumor CTL immunity are expected to have a greater protective potential in cancer, particularly to control highly heterogeneous tumors and metastatic disease.

## Methods

### Animals

C57BL/6/J wild-type (CD45.2), B6.Cg-Thy1^a^/Cy Tg(TcraTcrb)8Rest/J (pmel-1), C57BL/6-Tg(TcraTcrb)1100Mjb/J (OT-I), CBy.SJL(B6)-Ptprc^a^/J (CD45.1), B6.129S2-Cd207^tm3(DTR/GFP)Mal^/J (Langerin-DTR) mice were purchased from Jackson Laboratories, kept at the animal facility of Fundación Ciencia & Vida and maintained according to the “Guide to Care and Use of Experimental Animals, Canadian Council on Animal Care”. This study was carried out in accordance with the recommendations of the “Guidelines for the welfare and use of animals in cancer research, Committee of the National Cancer Research Institute”. All procedures complied with all relevant ethical regulations for animal research and were approved by the “Committee of Bioethics and Biosafety” of Fundación Ciencia & Vida. Blinding or randomization strategies were done whenever it was possible, no animals were excluded from the analysis and male and female mice were used indistinctly. Mice were allocated randomly in the different experimental procedures.

### Intravenous transfer of CD8+ T cells

Naïve CD45.1^+^ OTI and CD90.1^+^ pmel-1 TCR-transgenic CD8^+^ T cells were purified from secondary lymphoid organs of transgenic mice using the EasySep Mouse CD8^+^ T-Cell Enrichment Kit (StemCell Technologies, ref 19853). Mice were intravenously injected with 2.0-2.5 × 10^5^ cells in 100 µL of sterile PBS (ThermoFisher Scienfic, ref 10010023).

### Immunizations

One day after intravenous transfer of OTI CD8^+^ T cells, mice were intradermally immunized in the lower back skin with 40 µg of pVAX plasmid encoding a membrane bound form of chicken OVA (pOVA). Immediately, DNA electroporation was performed by placing a parallel needle array electrode (two rows of four 2 mm pins, 1.5 × 4 mm gaps; 47-0040, BTX electroporation systems) over the injected blebs to deliver the electric pulses (two 1125 V/cm, 0.05 ms pulses followed by eight 275 V/cm, 10 ms pulses) using the AgilePulse In Vivo System (BTX Molecular Delivery Systems, ref 47–0400N). Plasmids were purified using the NucleoBond PC10000 EF (Macherey-Nagel, ref 740548) or the NucleoBond XtraMidi EF (Macherey-Nagel, ref 740420.50).

### CD8^+^ T-cell depletion

At least 4 weeks after vaccination, mice were intraperitoneally injected with three doses of 20 µg of rat monoclonal anti-CD8 antibody (BioXCell, clone YTS169.4, ref BE0117) in consecutive days.

### Preparation of tissue cell suspensions

Inguinal lymph nodes were mechanically disaggregated and digested in 1 mL non-supplemented RPMI 1640 medium (ThermoFisher Scientific, ref 61870-036) containing 5 mg/mL of collagenase type IV (Gibco, ref 17104019) and 5 µg/mL of DNAse I (AppliChem, ref A3778,0010) for 30 min at 37 °C. Single-cell suspension was obtained using a 70 μm cell strainer (BD Falcon, ref 352350). For skin preparations, vaccinated skin was excised, cut in small fragments and digested in 1 mL non-supplemented RPMI 1640 medium (ThermoFisher Scientific, ref 61870-036) containing 5 mg/mL of collagenase type IV (Gibco, ref 17104019) and 5 µg/mL of DNAse I (AppliChem, ref A3778,0010) for 30 min at 37 °C, skin pieces were then disaggregated mechanically using microscope slides with ground edges (Sail Brand, ref 7105) and single-cell suspension was obtained using a 70 μm cell strainer (BD Falcon, ref 352350) followed by a second digestion with 1 mL of supplemented of RPMI 1640 medium containing 5 µg/mL of DNAse I (AppliChem, ref A3778,0010) during 5 min on ice.

### Flow cytometry staining

Cells were incubated 10 min with the TruStain fcX (clone 93) washed and incubated with the antibodies during 20 minutes followed by two washes with PBS. Monoclonal antibodies specific for mouse molecules were purchased from Biolegend: CD3-FITC (clone 17A2), CD3-APC (clone 17A2), CD3-PerCp/Cy5.5 (clone 17A2), CD8-Brillant Violet 421 (clone 53-6.7), CD45-PE (clone 30-F11), CD45-PerCP(clone 30-F11), CD45.1-PE/Cy7 (clone A20), CD45.1-FITC (clone A20), CD103-APC (clone 2E7), CD103-PerCP (clone 2E7), CD69-APC/Cy7 (clone H1.2F3), CD69-APC (clone H1.2F3), CD44-PerCP (clone IM7), IFN-γ-PE (clone XMG1.2), IFN-γ-APC (clone XMG1.2), TNF-α-APC/Cy7 (clone MP6-XT22), CD11b-FITC (clone M1/70), CD207-PE (clone 4C7), XCR1-APC (clone ZET), XCR1-PerCP-Cy5.5 (clone ZET), CD11c-PE/Cy7 (clone N418), MHCII-APC/Cy7 (clone M5/114.15.2), CD24-PerCP-Cy5.5 (clone M1/69), CD80-APC (clone 16-10A1), CD80-PE/Cy7 (clone 16-10A1), CD86 Brilliant Violet 421 (clone GL-1), CCR7-PE/Cy7 (clone 4B12), IL-2-PE/Cy7 (clone JES6-5H4) IL-12/23-APC (clone C15.6), granzyme B-APC (clone GB11) and viability dye Zombie Aqua (ref 423101). Samples were acquired in a FACSCanto II cytometer (BD Bioscience) and data were analyzed using FlowJo version X.0.7 (Tree Star, Inc.). Gating strategies for all flow cytometry experiments are shown in Supplementary Fig. 4.

### Ex vivo intracellular cytokine staining

Inguinal lymph nodes were obtained 12 days after the tumor challenge and CD8^+^ T cells were stimulated ex vivo with the gp100_(25-33)_ peptide (KVPRNQDWL, synthesized at Genscript) for 8 h. Brefeldin A (1 µg/mL, Sigma–Aldrich, ref B6542) was added during the last 6 h. In the case of neo-epitopes MUT1 (SIIVFNLL), MUT2 (AQLANDVVL) and MUT3 (ASMTNELM), stimulation was carried out during 20 h. Brefeldin A (1 µg/mL, Sigma–Aldrich, ref B6542) was added during the last 4 h. Intracellular staining was performed using the Cytofix/Cytoperm Fixation/Permeabilization solution set (BD Biosciences, ref 554714) according to the manufacturer’s instructions.

### In vivo Trm cell stimulation and intracellular cytokine staining

OVA-specific Trm cells were stimulated by intradermal injection with 20 µg of OVA_(257-264)_ peptide (SIINFEKL) or control SURV_20-28_ peptide (ATFKNWPFL) diluted in PBS near to the vaccination site. Mice were sacrificed 6, 24 or 48 h later and lymph nodes and skin were analyzed as described above. In the case of intracellular cytokine staining of skin Trm cells, 20 µg of brefeldin A was co-injected with the peptides. In the case of intracellular IL-12 staining of skin DCs, brefeldin A was injected after 24 h and analysis was performed 4 h later.

### Diphteria toxin administration

To deplete langerin-expressing DCs, Langerin-DTR mice received 1 µg of diphtheria toxin (Sigma–Aldrich, ref D0564 1MG) by intravenous injection in the tail vein. In the experiments where DC depletion was continuously maintained, mice received 0.35 µg of diphtheria toxin intraperitoneally every 3 days.

### Cell lines

Mouse melanoma cell line B16F10 (ATCC CLR-6475) was obtained from American Type Culture Collection. MC38 tumor cells were kindly provided by Dr. Burkhard Becher (University of Zurich, Switzerland) to Dr. Sergio A. Quezada. B16F10-OTIx5-ZsGreen (B16F10-OTI) and MC38-OTIx5-ZsGreen (MC38-OTI) cells were generated by lentiviral transduction of B16F10 cell line with the pLVX-OTIx5-ZsGreen vector encoding the OTI epitope minigene fused to ZsGreen^11^. B16F10 and MC-38 cell lines were cultured in complete RPMI 1640 (ThermoFisher Scientific, ref 61870-036) and DMEM (HyClone, ref SH30081.02) media, respectively, supplemented with penicillin, streptomycin (ThermoFisher Scientific, ref 15140122), non-essential amino acids (ThermoFisher Scientific, ref 11140050), sodium pyruvate (ThermoFisher Scientific, ref 11360070) and 10% of heat-inactivated fetal bovine serum (ThermoFisher Scientific, ref 10437010) in a humidified incubator at 37 °C with 5% CO_2_. All cell lines were routinely tested for mycoplasm contamination.

### Tumor challenge

Mice were injected intradermally in the lower back skin close to the vaccination site with 50 μL of PBS containing 1 × 10^6^ of tumor cells. Tumor growth was monitored by measuring perpendicular tumor diameters with calipers. Tumor volume was calculated using the following formula: V = (D x d^2^)/2 where V is the volume (mm^3^), D is larger diameter (mm) and d is smaller diameter (mm). Mice were sacrificed when moribund or when the mean tumor diameter was ≥15 mm,) according to the approved ethical protocol. When indicated, mice were re-challenged with 1 × 10^6^ B16F10 or MC38 cells in the contralateral site. For intravenous re-challenge 1 × 10^6^ of B16F10 melanoma cells in 200 ul of PBS were inoculated through the tail vein. Mice were sacrificed two weeks later and lungs were obtained, washed in PBS and stored in 3 mL of Fekete´s solution. Lung foci quantification was performed taking pictures of the lungs on both sides (Canon EOS rebel T5) followed by quantification of dark melanoma foci.

### RNA sequencing analysis

Upper quartile normalized RSEM expected RNA transcript counts and clinical data^47^ from The Cancer Genome Atlas (TCGA) project were downloaded from the National Cancer Institute GDC PanCanAtlas project website (https://gdc.cancer.gov/about-data/publications/pancanatlas) and cutaneous melanoma cases (SKCM) filtered. Trm cell and DC gene signatures were previously described by Charoetntong et al. and Savas et al.^35,45^. A tumor-infiltrating T-cell signature was used as previously described by Danaher et al.^48^. Non-protein coding genes were removed from these signatures for consistency with TCGA data. For each signature, enrichment scores were calculated by taking the mean log_10_ + 1 normalized expression of each gene, followed by z-score transformation. The correlation between Trm cell and DC gene signatures was evaluated by Pearson correlation.

### Statistical analysis

Statistical analysis was performed using Graphpad Prism software (Graphpad Software Inc.). RNA sequencing and survival analyses were carried out in the R statistical programming environment. Mann-Whitney unpaired tests were performed between relevant groups. Statistical analyses for tumor growth was performed using two-way ANOVA Bonferroni post-hoc test. Error bars in figures indicate the mean plus SEM. Survival analysis by Cox regression was carried out with the “survival” package and Kaplan-Meier survival curves were drawn using the “survminer” package with patients grouped on the median value of each variable tested and with log-rank p values reported. Overall p value <0.05 was considered statistically significant; *p ≤ 0.05, **p ≤ 0.01, ***p ≤ 0.001 and ****p ≤ 0.0001.

### Reporting summary

Further information on research design is available in the Nature Research Reporting Summary linked to this article.

## Supplementary information

Supplementary Information

Peer Review File

Reporting Summary

## Data Availability

The data that support the findings of this study are available from the authors on reasonable request.
