# Supplementary material for: Tissue-resident memory CD8+ T cells amplify anti-tumor immunity by triggering antigen spreading through dendritic cells
Source: Nat Commun. 2019 Sep 27;10:4401. doi: 10.1038/s41467-019-12319-x (PMC6765014; doi:10.1038/s41467-019-12319-x)
Supplement: Supplementary file 1 — Supplementary Information [file 41467_2019_12319_MOESM1_ESM.pdf]

Supplementary information

Resident memory CD8^{+} T cells amplify anti-tumor immunity by triggering antigen spreading through dendritic cells

Menares et al.

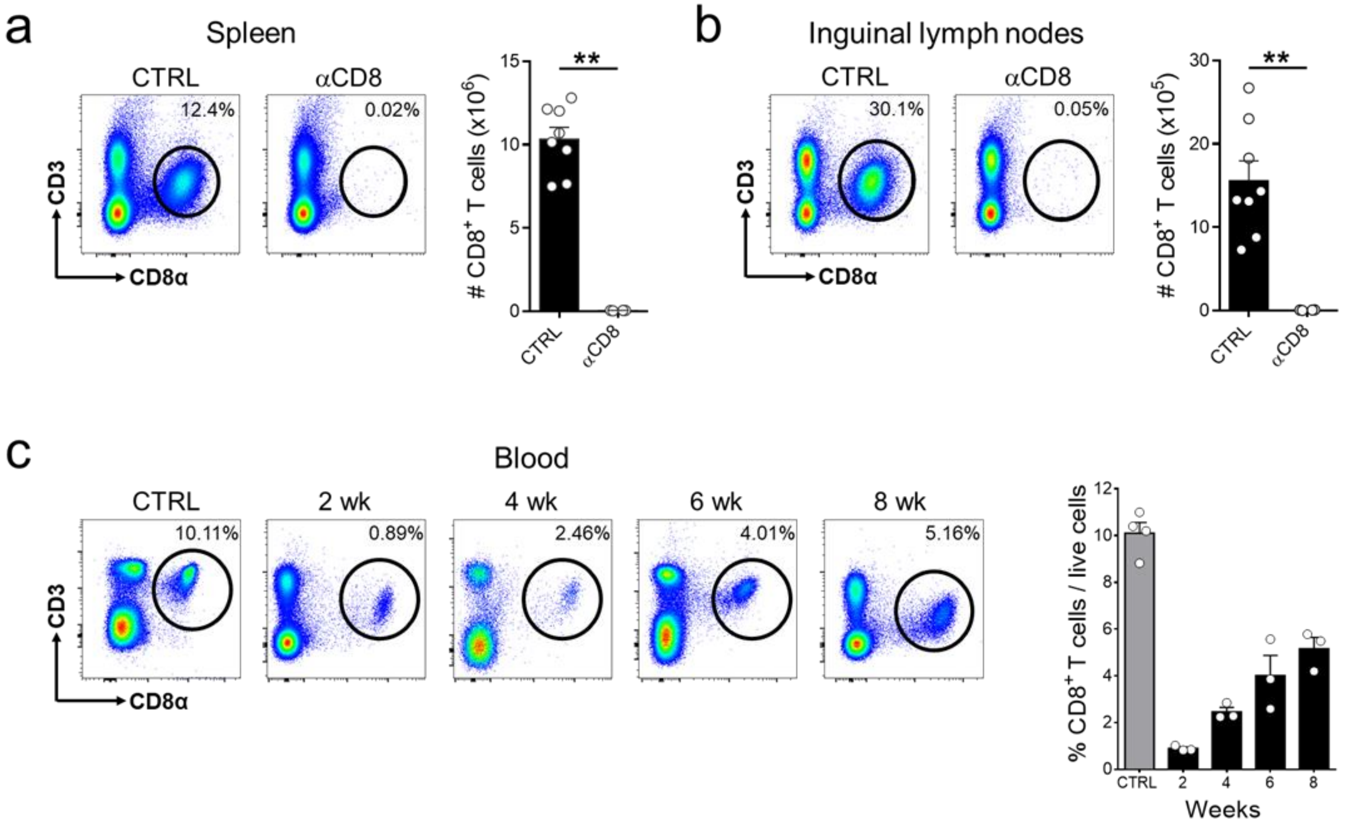

Supplementary Figure 1. Antibody-dependent depletion and replenishment of CD8$^+$ T cells. C57BL/6 naïve mice were injected intraperitoneally with three doses (20 µg each) of isotype control (CTRL) or anti-CD8 antibodies in three consecutive days. Three days later, spleen and inguinal lymph nodes were analyzed by flow cytometry to evaluate the frequencies of CD8$^+$ T cells. To evaluate the replenishment of CD8$^+$ T cells, blood samples were analyzed every two weeks. (a-c) Representative pseudocolor dot-plots showing the expression of CD3 and CD8 in total live cells and the quantification of CD8$^+$ T cells. (c) Analysis performed in blood at different timepoints. Pooled data from two independent experiments, n=4-8 for (a-b), and from one experiment n=3-4 for (c). Bars are the mean ± SEM ** $p < 0.01$, by Mann-Whitney unpaired test.

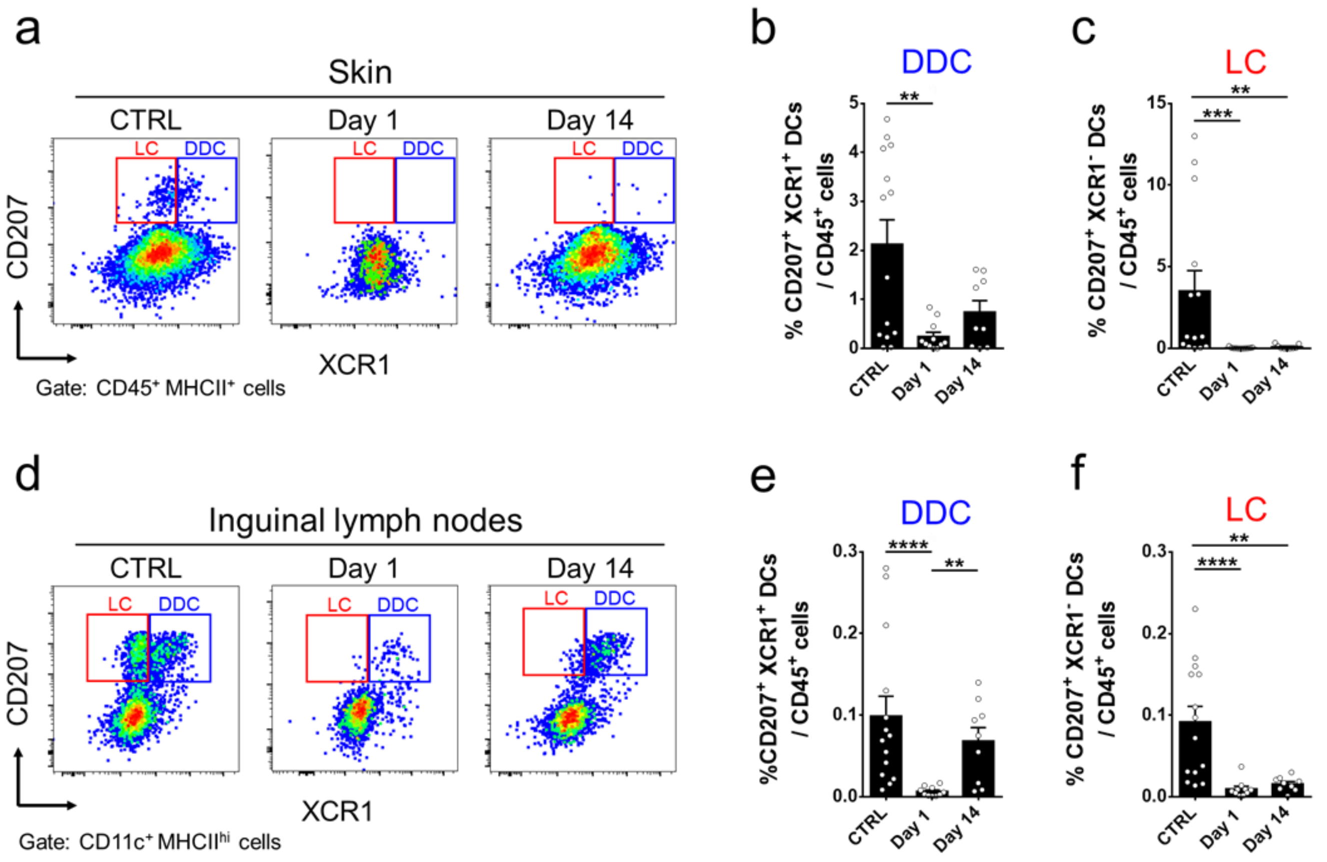

Supplementary Figure 2. Skin DC depletion in Langerin-DTR mice. Langerin-DTR mice were intravenously inoculated with 1 µg of diphtheria toxin (DTx) followed by analysis of langerin/CD207 and XCR1 in DCs present in skin and inguinal lymph nodes 1 and 14 days later. Among the population of MHC II^{+} (skin, a-c) or CD11c^{+}MHCII^{high} (lymph nodes, d-f) DDC and LC were defined as CD207^{+}XCR1+$^{-}$ and CD207+XCR1-, respectively. (a, d) Representative dot-plots showing DC subsets. (b-c, e-f) Graphs showing quantification of DDC (b, e) and LC (c, f), as frequency of total CD45^{+} cells. Pooled data from four independent experiments, n=14 mice in control group, n=11 mice in Day 1 group, and n=9 in Day 14 group. Bars are the mean ± SEM, ** p <0.01, *** p <0.01, **** p <0.0001 by Mann-Whitney unpaired test.

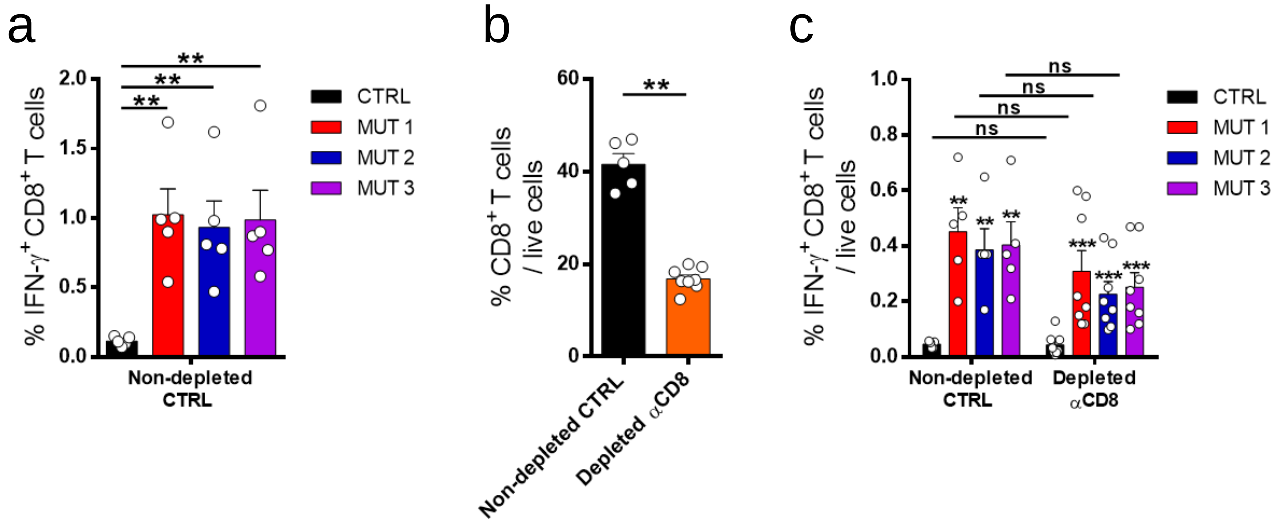

Supplementary Figure 3. Trm cell-induced spreading of CTL responses in non-depleted mice. C57BL/6 mice were vaccinated to generate OVA-specific circulating and skin-resident memory CD8^{+} T cells. At least four weeks later, mice were either non-depleted or depleted of circulating CD8^{+} T cells by administration of isotype control or anti-CD8 antibodies, respectively. Then, mice were challenged intradermally with MC38-OTI cells. In the case of depleted mice, tumor challenge was performed eight weeks after antibody depletion. Twelve days after tumor challenge, CD8^{+} T cells were analyzed in draining lymph nodes after *ex vivo* stimulation with control (CTRL) or neo-epitope peptides (MUT 1, MUT 2 and MUT 3) followed by intracellular cytokine staining. (a) Percentage of IFN-γ-producing CD8^{+} T cells in non-depleted mice. (b) Analysis of total CD8+ T cells. (c) Frequencies of IFN-γ-producing CD8^{+} T cells relative to total live cells. Data correspond to one experiment with n=5 mice for the Non-depleted group. Pooled data from two independent experiments n=8 mice for the Depleted group. Bars are the mean ± SEM. ** $p \leq 0.01$ by Mann-Whitney unpaired test.

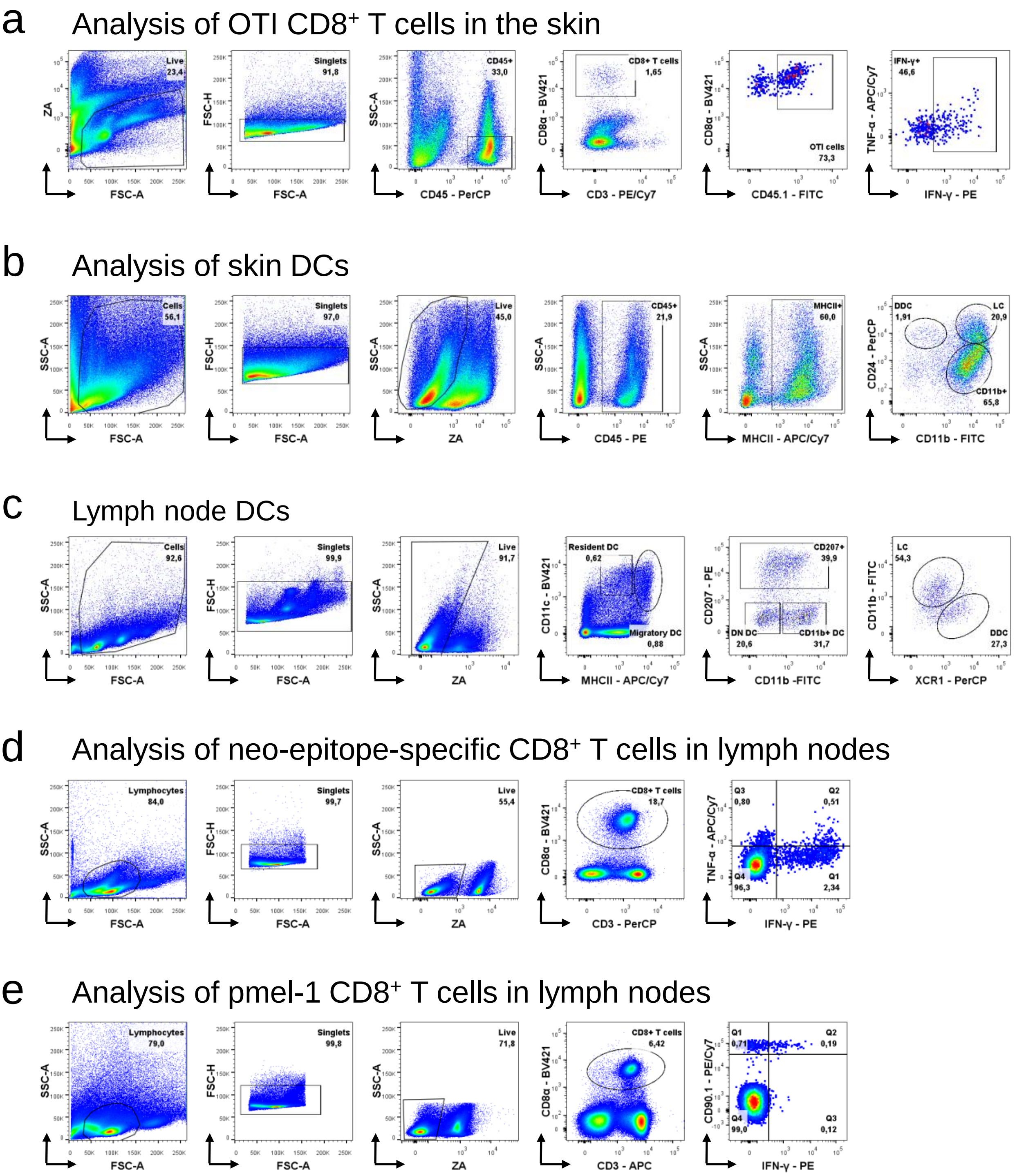

Supplementary Figure 4. Gating strategies used in the manuscript. (a) Analysis of OTI CD8$^+$ T cells in the skin, used in figure 1a-b. (b) Analysis of skin DCs, used in figure 1c-g. (c) DC analysis in lymph node used in figure 2a-c. (d) Analysis of neo-epitope-specific CD8$^+$ T cells in lymph nodes, used in figure 3b-g. (e) Analysis of pmel-1 CD8$^+$ T cells in lymph nodes, used in figure 4b-f.
