# Supplementary material for: Tissue-resident memory CD8+ T cells amplify anti-tumor immunity by triggering antigen spreading through dendritic cells
Source: Nat Commun. 2019 Sep 27;10:4401. doi: 10.1038/s41467-019-12319-x (PMC6765014; doi:10.1038/s41467-019-12319-x)
Supplement: Supplementary file 3 — Reporting Summary [file 41467_2019_12319_MOESM3_ESM.pdf]

nature research

Corresponding author(s): Alvaro Lladser

Last updated by author(s): Aug 22, 2019

Reporting Summary

Nature Research wishes to improve the reproducibility of the work that we publish. This form provides structure for consistency and transparency in reporting. For further information on Nature Research policies, see Authors & Referees and the Editorial Policy Checklist.

Statistics

For all statistical analyses, confirm that the following items are present in the figure legend, table legend, main text, or Methods section.

n/a	Confirmed	
☐	☒	The exact sample size (*n*) for each experimental group/condition, given as a discrete number and unit of measurement
☐	☒	A statement on whether measurements were taken from distinct samples or whether the same sample was measured repeatedly
☐	☒	The statistical test(s) used AND whether they are one- or two-sided<br>*Only common tests should be described solely by name; describe more complex techniques in the Methods section.*
☐	☒	A description of all covariates tested
☐	☒	A description of any assumptions or corrections, such as tests of normality and adjustment for multiple comparisons
☐	☒	A full description of the statistical parameters including central tendency (e.g. means) or other basic estimates (e.g. regression coefficient) AND variation (e.g. standard deviation) or associated estimates of uncertainty (e.g. confidence intervals)
☒	☐	For null hypothesis testing, the test statistic (e.g. F, t, r) with confidence intervals, effect sizes, degrees of freedom and P value noted<br>*Give P values as exact values whenever suitable.*
☒	☐	For Bayesian analysis, information on the choice of priors and Markov chain Monte Carlo settings
☒	☐	For hierarchical and complex designs, identification of the appropriate level for tests and full reporting of outcomes
☐	☒	Estimates of effect sizes (e.g. Cohen's d, Pearson's r), indicating how they were calculated

Our web collection on statistics for biologists contains articles on many of the points above.

Software and code

Policy information about availability of computer code

Data collection	*Provide a description of all commercial, open source and custom code used to collect the data in this study, specifying the version used OR state that no software was used.*
Data analysis	FlowJo version X.0.7 (Tree Star, Inc.) were used to perform flow cytometry analysis.<br>Graphpad Prism 6 software (Graphpad Software Inc.) were used to perform general statistical analysis.

For manuscripts utilizing custom algorithms or software that are central to the research but not yet described in published literature, software must be made available to editors/reviewers. We strongly encourage code deposition in a community repository (e.g. GitHub). See the Nature Research guidelines for submitting code & software for further information.

Data

Policy information about availability of data

All manuscripts must include a data availability statement. This statement should provide the following information, where applicable:
- Accession codes, unique identifiers, or web links for publicly available datasets
- A list of figures that have associated raw data
- A description of any restrictions on data availability

The data that support the findings of this study are available from the corresponding author upon reasonable request.

October 2018

Field-specific reporting

Please select the one below that is the best fit for your research. If you are not sure, read the appropriate sections before making your selection.

☒ Life sciences ☐ Behavioural & social sciences ☐ Ecological, evolutionary & environmental sciences

For a reference copy of the document with all sections, see nature.com/documents/nr-reporting-summary-flat.pdf

Life sciences study design

All studies must disclose on these points even when the disclosure is negative.

Sample size	No statistical test was used to pre-determine sample size.
Data exclusions	Outliers were identified by Grubb´s Test. Outliers were discarded from fig. 1d, 1f, 1g, 2b, 2c, 4c, suppl. fig. 2e and suppl. fig 2f.
Replication	The numbers of repeats for experiment was described in each figure legends.
Randomization	No pre-established selection criteria for mice were used.<br>No animals were excluded from the analysis and male and female mice were used indistinctly.
Blinding	The investigators were not blinded during experiments.

Reporting for specific materials, systems and methods

We require information from authors about some types of materials, experimental systems and methods used in many studies. Here, indicate whether each material, system or method listed is relevant to your study. If you are not sure if a list item applies to your research, read the appropriate section before selecting a response.

Materials & experimental systems

n/a	Involved in the study	
☐	☒	Antibodies
☐	☒	Eukaryotic cell lines
☒	☐	Palaeontology
☐	☒	Animals and other organisms
☒	☐	Human research participants
☐	☒	Clinical data

Methods

n/a	Involved in the study	
☒	☐	ChIP-seq
☐	☒	Flow cytometry
☒	☐	MRI-based neuroimaging

Antibodies

Antibodies used

CCR7-PE/Cy7(clone 4B12) 120124 Biolegend
CD103-APC (clone 2E7) 121414 Biolegend
CD103-PerCP (clone 2E7) 121416 Biolegend
CD11b-FITC (clone M1/70) 101206 Biolegend
CD11c-PE/Cy7 (clone N418) 117318 Biolegend
CD207-PE (clone 4C7) 144204 Biolegend
CD24-PerCP-Cy5.5 (clone M1/69) 101824 Biolegend
CD3-APC (clone 17A2) 100236 Biolegend
CD3-FITC (clone 17A2) 100204 Biolegend
CD3-PerCp/Cy5.5 (clone 17A2) 100218 Biolegend
CD44-PerCP (clone IM7) 103036 Biolegend
CD45.1-FITC (clone A20) 110706 Biolegend
CD45.1-PE/Cy7 (clone A20) 110730 Biolegend
CD45-PE (clone 30-F11) 103106 Biolegend
CD45-PerCP(clone 30-F11) 103130 Biolegend
CD69-APC (clone H1.2F3) 104514 Biolegend
CD69-APC/Cy7 (clone H1.2F3) 104526 Biolegend
CD80-APC (clone 16-10A1) 104714 Biolegend
CD80-PE/Cy7 (clone 16-10A1) 104734 Biolegend
CD86 Brilliant Violet 421 (clone GL-1) 105032 Biolegend
CD8-Brillant Violet 421 (clone 53-6.7) 100737 Biolegend
Granzyme B-APC (clone GB11) GRB05 Invitrogen
IFN-γ-APC (clone XMG1.2) 505810 Biolegend
IFN-γ-PE (clone XMG1.2) 505808 Biolegend
IL-12/23-APC (clone C15.6) 505205 Biolegend

	IL-2-PE/Cy7 (clone JES6-5H4) 503832 Biolegend<br>MHCII-APC/Cy7 (clone M5/114.15.2) 107628 Biolegend<br>TNF-α-APC/Cy7 (clone MP6-XT22) 506344 Biolegend<br>XCR1-APC (clone ZET) 148206 Biolegend<br>XCR1-PerCP-Cy5.5 (clone ZET) 148208 Biolegend
Validation	Antibodies were purchased directly from BioLegend (https://www.biolegend.com/en-us/reproducibility) excepting Granzyme B-APC (clone GB11) GRB05 Invitrogen (https://www.thermofisher.com/antibody/product/Granzyme-B-Antibody-clone-GB11-Monoclonal/GRB05).

Eukaryotic cell lines

Policy information about cell lines

Cell line source(s)	B16F10 cells was obtained from American Type Culture Collection.<br>MC38 cells were kindly provided by Dr. Burkhard Becher (University of Zurich, Switzerland) to Dr. Sergio A. Quezada.<br>The transduced cell lines were gendered as described in the materials and methods section.
Authentication	*Describe the authentication procedures for each cell line used OR declare that none of the cell lines used were authenticated.*
Mycoplasma contamination	The cell lines were frequently checked for mycoplasma by PCR.
Commonly misidentified lines<br>(See ICLAC register)	None of cell lines belongs to misidentified cell lines.

Animals and other organisms

Policy information about studies involving animals; ARRIVE guidelines recommended for reporting animal research

Laboratory animals	C57BL/6/J wild-type (CD45.2), B6.Cg-Thy1a/Cy Tg(TcraTcrb)8Rest/J (pmel-1), C57BL/6-Tg(TcraTcrb)1100Mjb/J (OT-I), CBy.SJL(B6)-Ptprca/J (CD45.1), B6.129S2- Cd207 tm3(DTR/GFP)Mal /J (Langerin-DTR) mice were purchased from Jackson Laboratories, kept at the animal facility of Fundación Ciencia & Vida. The mice were maintained at a temperature of 26 ° -28 ° C, in cycles of light and dark of 12 hours, with water and food ad libitum. When starting the experiments the mice were between 8 and 12 weeks old.
Wild animals	
Field-collected samples	*For laboratory work with field-collected samples, describe all relevant parameters such as housing, maintenance, temperature, photoperiod and end-of-experiment protocol OR state that the study did not involve samples collected from the field.*
Ethics oversight	Animales were maintained according to the “Guide to Care and Use of Experimental Animals, Canadian Council on Animal Care”. This study was carried out in accordance with the recommendations of the “Guidelines for the welfare and use of animals in cancer research, Committee of the National Cancer Research Institute”. The protocol was approved by the “Committee of Bioethics and Biosafety” of Fundación Ciencia & Vida.

Note that full information on the approval of the study protocol must also be provided in the manuscript.

Clinical data

Policy information about clinical studies

All manuscripts should comply with the ICMJE guidelines for publication of clinical research and a completed CONSORT checklist must be included with all submissions.

Clinical trial registration	*Provide the trial registration number from ClinicalTrials.gov or an equivalent agency.*
Study protocol	*Note where the full trial protocol can be accessed OR if not available, explain why.*
Data collection	Upper quartile normalized RSEM expected RNA transcript counts and clinical data [43] from The Cancer Genome Atlas (TCGA) project were downloaded from the National Cancer Institute GDC PanCanAtlas project website (https://gdc.cancer.gov/about-data/publications/pancanatlas) and cutaneous melanoma cases (SKCM) filtered.
Outcomes	*Describe how you pre-defined primary and secondary outcome measures and how you assessed these measures.*

Flow Cytometry

Plots

Confirm that:

☐ The axis labels state the marker and fluorochrome used (e.g. CD4-FITC).

☐ The axis scales are clearly visible. Include numbers along axes only for bottom left plot of group (a 'group' is an analysis of identical markers).

☒ All plots are contour plots with outliers or pseudocolor plots.

☒ A numerical value for number of cells or percentage (with statistics) is provided.

Methodology

Sample preparation	All the tissue preparation protocols for flow cytometry were described in the materials and methods section.
Instrument	FACSCanto II cytometer (BD Bioscience)
Software	FlowJo version X.0.7 (Tree Star, Inc.) were used to perform flow cytometry analysis.
Cell population abundance	*Describe the abundance of the relevant cell populations within post-sort fractions, providing details on the purity of the samples and how it was determined.*
Gating strategy	Figures show the gatting strateggies.

☒ Tick this box to confirm that a figure exemplifying the gating strategy is provided in the Supplementary Information.
