# Supplementary material for: Tissue-resident memory CD8+ T cells amplify anti-tumor immunity by triggering antigen spreading through dendritic cells
Source: Nat Commun. 2019 Sep 27;10:4401. doi: 10.1038/s41467-019-12319-x (PMC6765014; doi:10.1038/s41467-019-12319-x)
Supplement: Supplementary file 2 — Peer Review File [file 41467_2019_12319_MOESM2_ESM.docx]

Dr. Tanya Bondar
Senior Editor
Nature Communications

Dear Dr. Bondar,

We really appreciate the insightful and constructive revision of our manuscript. We performed additional experiments to address the concerns raised by the reviewers, including further documentation of Trm cell and DC phenotypes, as well as the expansion of tumor-specific CD8^+^ T cells of the endogenous repertoire. We have included these new data and modified the manuscript accordingly. All relevant changes were highlighted in yellow in the manuscript. Please find our answers (in blue) to specific Reviewer’s comments, outlined below point by point. We think that the revision has significantly improved the quality and impact of our manuscript and we hope that our efforts will be sufficient for publication in Nature Communications.

Sincerely,

Alvaro Lladser.

**Reviewer #1 (Remarks to the Author):** 

The authors propose that resident memory T cells promote the maturation and trafficking to draining lymph node of dendritic cells and favour the expansion of anti-tumor CD8+T cells. 

1) From the results reported, it is clear that there is a correlation between the activation of skin derived CD8+T cells and the activation of dendritic cells and an increase of anti-tumor immunity. However, the direct link between the activation of resident memory CD8+T cells (TRM) in skin and the maturation of dendritic cells or the expansion of CD8+T cells is less convincing. Indeed, even if TRM are the dominant CD8+T cells in the tissue, they are not the only local CD8+T cells. In figure 1a, they gated on CD8+T cells and conclude that the TRM produce cytokines. Effector memory CD8+T cells are also often present in the tissue or in the tumor microenvironment.

The respective role of TRM or effector memory T cells may be assessed by using the FTY720 inhibitor, anti-TGFb during the priming or parabiosis experiments (Enamorado M et al Nat Commun 2017, Nizard M et al Nat Commun 2017)

We realized that the way we presented the data may confuse the reader on whether CD8+ T cells present in the skin after antibody-depletion are actually Trm cells. We and other have demonstrated that anti-CD8 antibody depletion efficiently eliminates the circulating compartment, including memory and effector CD8+ T cell subsets, in lymphoid and non-lymphoid tissues, while sparing the skin resident compartment (Supplementary Figure 1) [1]. To make our point clear, we have included a supplementary figure (Supplementary Figure 1) showing efficient CD8^+^ T cell depletion in spleen, lymphoid organs and blood after anti-CD8 antibody administration. We have also added a representative dot plot in Figure 1 showing that the OVA-specific CD8^+^ T cells that remain in the skin after antibody depletion have a canonical CD69^+^CD103^+^ Trm phenotype. Now, it becomes more clear that antigen-specific activation of Trm cells, characterized by the production of IFN-γ and TNF-α, triggers maturation of dendritic cells and the subsequent expansion of CD8^+^ T cell responses against tumor-derived antigens.

S1PR1 antagonist FTY720 has been broadly used for restraining circulating CD8^+^ T cells in lymphoid organs, in this way, limiting their recirculation and their migration to peripheral tissues, such skin [2-4]. However, we have observed that CD8^+^ T cell depletion is far more efficient than FTY720 at reducing circulating CD8^+^ T cell numbers in peripheral blood (Reviewers Figure 1, at the end of this document). In addition, FTY720 is not specific for CD8^+^ T cells, as it also affects CD4^+^ T cell migration (Reviewers Figure 1). Moreover, emerging evidence indicates that FTY720 may affect tumor growth by directly suppressing tumor cell proliferation, impairing T cell function or interfering with the ability of T cells to infiltrate tumors [5-9][10]. Therefore, we consider that CD8^+^ T depletion represents a better model than FTY720 to rule-out the contribution of the circulating compartment.

[REDACTED]

The other model suggested by the Reviewer to dissect the contribution of the circulating and resident compartments is parabiosis. However, it would take more time than reasonable for the timeframe of this revision to acquire the expertise to carry out these experiments in our laboratory (which includes obtaining the ethical permit, establishment of this model, and running the experiments needed for this manuscript. Instead, we decided to address the contribution of the circulating compartment by comparing the expansion of secondary CD8^+^ T cell responses induced by either only resident (OTI WT depleted) or both circulating and resident (OTI WT non-depleted) compartments. As observed in Supplementary Figure 3c, the responses against tumor-derived antigens in the presence of both circulating and resident memory OVA-specific CD8+ T cell compartments were comparable to the responses observed in mice with only resident memory OVA-specific CD8+ T cell compartment (Figure 3, Supplementary Figure 3). These results indicate that the circulating compartment is dispensable and that most of the antigen spreading capacity lies on the resident memory compartment.

2) It is a pity that the authors used the adoptive transfer of an anti-tumor CD8+T cell clone to show that local CD8+T cells play a role in the spreading of anti-tumor response. What about the role of local CD8+T cells in the expansion of endogenous anti-tumor CD8+T cells not directed against Ova.

We fully agree with the reviewers about the importance of demonstrating the expansion of endogenous anti-tumor CD8+ T cells triggered by Trm cell-mediated tumor rejection. Given that endogenous anti-tumor CD8+ T cell responses are skewed towards neo-antigens [13], we decide to test CTL expansion using the MC38 cell line that has been reported to express multiple neo-antigens product of missense mutations [14]. Mice bearing OVA-specific Trm cells were depleted of circulating CD8+ T cells and let to replenish the endogenous repertoire during 8 weeks before challenging them with MC38-OTI. To test antigen spreading, lymph node cells were stimulated with three different validated neo-epitopes: Mut 1 (SIIVFNLL from Dpagt1), Mut 2 (AQLANDVVL from Reps1) and Mut 3 (ASMTNMELM from Adpgk) (Figure 3). Interestingly, we observed specific CTL responses against the three neo-antigens tested, detected as IFN-γ-producing CD8^+^ T cells (Figure 3b,c). These responses were not observed in mice bearing OVA-Trm cells challenged with parental MC38 cell line not expressing OTI peptide, or in mice with no Trm challenged with MC38-OTI (Figure 3c). Neo-epitope-specific CD8^+^ T cells also display higher expression of effector molecules such as IL-2, TNF-α and granzyme B (Figure 3e-g), indicating that they acquired a functional cytotoxic phenotype. Accordingly, mice bearing neo-epitope-specific CTLs also rejected a re-challenge with parental MC38 cell line (Figure 3h-k). These results support the notion that Trm cells are able to trigger the spreading of CTL responses against multiple highly relevant tumor-derived antigens with the potential to protect against tumors that are no longer recognized by Trm cells. These new data have been included as new Figure 3 and mentioned in the Results and Discussion sections.

3) The authors depleted systemic CD8+T cells to eliminate their potential role compared to Trm. However, how long did this depletion occur, as some experiments went on more than 3 weeks after this depletion. The authors showed that Trm are protected from this depletion, but what about the CD8+T cells in the lymph node.

We agree with the reviewer that is important to clarify how efficient is antibody-dependent CD8^+^ T cell depletion in lymphoid organs and the kinetics replenishment of circulating CD8^+^ T cells. For this reason, we included Supplemmentary Figure 1 showing that CD8^+^ T cell depletion is nearly complete in spleen and inguinal lymph nodes (Supplemmentary Figure 1a,b), as well as non-lymphoid tissues [1]. In consequence, acute antibody depletion also efficiently eliminates circulating memory and effector OVA-specific CD8^+^ T cells generated after vaccination, while sparing OVA-specific Trm cells [1]. Then, circulating CD8^+^ T cells with new specificities are gradually replenished reaching nearly 50% of the level present in non-depleted mice after 8 weeks, as observed in blood (Supplemmentary Figure 1c). In other words, acute antibody depletion allows to reset the circulating compartment in terms of specificities by eliminating antigen-experienced CD8^+^ T cells, and allows to dissect the function of the resident memory compartment by ruling out the contribution of the circulating compartment.

4) How did the author explain that the administration of a CD8+T cell clone is not activated when recognizing their target on tumor cells without the presence of local CD8+T cells ?

This is a very relevant question. In our melanoma model, the status of pmel-1 CD8+ T cells is analyzed twelve days later after challenge. At this moment, B16F10-OTI cells have already been rejected by Trm cells, triggering the maturation and migration to lymph nodes of dermal DCs, probably carrying tumor-derived antigens, to initiate secondary responses. Whereas, B16F10 tumors are growing, which are known to be poorly immunogenic. As proof of this, it was shown that B16F10 tumors can normally grow in TCR-transgenic pmel-1 mice, whose virtually all CD8+ T cells are specific against the gp100_(25–33)_ epitope, unless CD8+ T cells are exogenously primed [15]. These results probably reflect a limited infiltration of cross-presenting DCs in B16F10 tumors, resulting in inefficient activation of melanoma-reactive CD8^+^ T cells [16]. Consistently, we observe no significant expansion of pmel-1 CD8+ T cells in lymph nodes, with the majority displaying a naïve phenotype (Figure 4). As a complement to these data, a more immunogenic MC38 model was used (Figure 3). Similar conclusions can be depicted from this model, where no significant expansion of CD8^+^ T cells against tumor-derived neo-antigens was observed in mice bearing OVA-speciic Trm cells and challenged with control MC38, in contrast with the marked expansion observed with MC38-OTI. Moreover, in these experiments we included an additional control group that helps to address this important issue. We used mice that did not have Trm cells and were challenged with MC38-OTI. In this mice, we observed no significant expansion of neo-epitope-specific CD8+ T cells, indicating that spontaneous antigen spreading is not efficiently produced and need to be facilitated by Trm cell-mediated tumor recognition.

**Reviewer #2 (Remarks to the Author):** 


This manuscript describes how a resident memory CD8 T cells (Trm) can interact with dermal DCs upon antigen specific activation. Due to this crosstalk they have observed melanoma rejection mediated through Trm CD8 T cells. The authors also show how dermal DC can induce new effector T cells via maturation by Trm CD8 cells, and according to their hypothesis can lead to epitope spreading. The overall outcome of this data is potential interesting, although so far, all tumour experiments address only tumour regression after vaccination, as the study is based on Trm already being present. No therapeutic models were incorporated. 

Major comments: 

- Figure 1: In order to make a robust phenotype of the resident T cells the authors should include at least a positive marker for resident cells as: CD103+ or Clec9A. (Collin et al, 2013).

As stated before, we understand that the way data are presented in Figure 1 may confuse the reader on whether CD8+ T cells present in the skin after antibody-depletion are actually Trm cells. We and other have demonstrated that anti-CD8 antibody depletion efficiently eliminates the circulating compartment, including memory and effector CD8+ T cell subsets, in lymphoid and non-lymphoid tissues, while sparing skin Trm cells [17] (Supplementary Figure 1). To make this clear, we have included a representative dot plot in Figure 1 showing that the OVA-specific CD8+ T cells that remain in the skin after antibody depletion have a canonical CD69+ CD103+ Trm phenotype.

- Figure 1: CD86 is not enough to evaluate the maturation status of DCs. Other costimulatory markers such as CD80, CD40, CD83 should be evaluated. In addition, intracellular staining of IL-12 should be performed to get infoemation on the functional status of the DCs activation.

We fully understand this concern raised by the reviewer. To address this issue, we have performed new experiments evaluating the expression of additional maturation markers, including CD80 and MHC class II, as well as intracellular staining of IL-12. As observed in updated Figure 1, Trm cell activation up-regulates the expression of all these maturation markers and IL-12 production in DDCs, as compared to other skin DC subsets (Figure 1d-g). These results allow us to conclude that Trm cell activation induces DDC maturation.

- Figure 2: For migratory DCs is necessary include markers for migration such as CCR7.

After the reviewer pointed this out, we performed additional experiments analyzing CCR7 in lymph nodes. Expectedly, the different migratory DC subsets show high expression of CCR7 in contrast to lymph-node-resident DCs (Figure 2a, right panel). Interestingly, DDCs have the highest levels of CCR7 among DC subsets.

- Figure 3: next to the use of the CD44 marker to determine the activated CD8 population, additional support may come by evaluating other factors such as TNF-a or GBZ.

Even if CD44-expressing CD8^+^ T cells normally display a differentiated cytotoxic phenotype, we sought to confirm this experimentally by analyzing the expression of effector molecules in CTLs expanded from the endogenous repertoire induced after MC38-OTI challenge (Figure 3). As expected, neo-epitope-specific IFN-γ-producing CD8^+^ T cells express higher levels of IL-2, TNF-α and granzyme b, as compared to bulk CD8^+^ T cell population (Figure 3e-g). Consistently, mice carrying neo-epitope-specific CD8^+^ T cells were able to reject a re-challenge with MC38 cells, which do not express OVA-derived peptides. These results support the conclusions that Trm cell-mediated rejection triggers the generation of secondary CTL responses against tumor-derived antigens with anti-tumor potential.

- Figure 4: The timeline for the analysis is not sufficient to evaluate the response of the trm therapy. All tumour experiments relate to tumour challenges after vaccination, since the basis of the study is the presence of Trm CD8. Since the current setup requires the generation of Trm, testing the role of Trm in a therapeutic setting where the tumour is present before vaccination is not possible. Obviously, the study would gain much in relevance when Trm cross talk and epitope spreading could also be demonstrated in a more therapeutic setting.

We fully understand this concern raised by the reviewer, however the timing of Trm cell formation is a major limitation to address this issue, not only for us but for other groups working in the field [18, 19]. We have been developing different strategies to test antitumor activity of Trm cells in more therapeutic settings, and then study antigen spreading. One of our attempts was the generation of B16F10 cells with tamoxifen-inducible expression of the OTI peptide. This would have allowed us to timely control antigen recognition by Trm cells when tumors have already grown. Unfortunately, B16F10-iOTI cells were rejected by mice bearing OVA-specific Trm cells before even inducing the expression of the OTI peptide (Reviewers Figure 3), which is probably the result of leaky expression of the OTI gene, as it has been previously suggested for tamoxifen-inducible constructs. We consider that despite not having a more therapeutic model available at this point, our work unveils a novel mechanism by which Trm cells can boost anti-tumor immunity via DCs. We envision that this mechanism may also be important in therapeutic settings but experimental evidence supporting this statement still remains to be seen.

Aside from this, how sure are the authors that the results are really due to epitope spreding? For example, the increased expansion Pmel cells could be solely due to DC maturation by Trm’s. It is known that B16.F10 cells start class I MHC expression only upon IFN-y stimulation and during the first challenge, ova peptide can induce IFN-y from Trm’s. Eventually, the expansion of Pmels could as well be due to increased antigen presentation by B16.F10 cells.

The concept of antigen spreading can be defined as the expansion of adaptive immune responses against secondary antigens that were not targeted by the original intervention or therapy. However, the mechanisms by which these secondary responses can be elicited are multiple and are not completely defined. In the context of this work, a plausible mechanism could involve Trm cell-promoted DC maturation and subsequent presentation of tumor-derived antigens for more efficient CD8^+^ T cell priming. It is also true that B16F10 cells up-regulate MHC class I expression after IFN-γ stimulation, which can derive from antigen-activated Trm cells. However, if expansion of pmel-1 CD8^+^ T cells would be due to increased antigen presentation by B16F10 cells, we should observe efficient CTL expansion in Lang-DTR mice (Figure 4e) regardless the status of skin DC subsets. In contrast, expansion of pmel-1 CD8^+^ T cells does not occur in the absence of dermal DCs (Continous DTx treatment). In this condition, Trm cells should still be able to recognize B16F10-OTI cells to produce IFN-γ and increase antigen presentation by B16F10 cells, but it does not result in CTL expansion. Our data indicate that dermal DCs are necessary for antigen spreading to occur. These cells could either carry tumor-derived antigens to lymph nodes and/or directly present these antigens to naïve CD8^+^ T cells. We have now addressed this in the Results section of the revised manuscript.

Additionally, it is hard to justify the transfer of Pmel cells while claiming antigen spreading. Accordingly, when the Pmel cells omitted in tumor model, no significant difference was seen in tumor growth or metastasis formation. The authors should instead check the endogenously generated gp100 specific T cells by commercially available tetramers or dextramers. Moreover, an in vivo cytotoxicity experiment performed in sequential vaccinations of only ova and ova+gp100 peptides can as well demonstrate the help provided by Trm cells. Unfortunately, in its current state, the data on antigen spreading are not conclusive. ???

We fully agree with the reviewers about the importance of demonstrating the expansion of endogenous anti-tumor CD8^+^ T cells triggered by Trm cell-mediated tumor rejection. Although we were not able to detect endogenous GP100-specific CD8^+^ T cell responses by dextramer staining (data not shown), we tested endogenous CTL expansion using the MC38 cell line that has been reported to express multiple neo-antigens product of missense mutations [14]. With this purpose, mice bearing OVA-specific Trm cells were depleted of circulating CD8^+^ T cells and let to replenish the endogenous repertoire during 8 weeks before challenging them with MC38-OTI. To test antigen spreading, lymph node cells were stimulated with three different validated neo-epitopes: Mut 1 (SIIVFNLL from Dpagt1), Mut 2 (AQLANDVVL from Reps1) and Mut 3 (ASMTNMELM from Adpgk) (Figure 3). Interestingly, we observed specific CTL responses against the three neo-antigens tested, detected as IFN-γ-producing CD8^+^ T cells (Figure 3b,c). These responses were not observed in mice bearing OVA-Trm cells challenged with parental MC38 cell line not expressing OTI peptide, or in mice with no Trm challenged with MC38-OTI (Figure 3c). Neo-epitope-specific CD8^+^ T cells also display higher expression of effector molecules such as IL-2, TNF-α and granzyme B (Figure 3e-g), indicating that they acquired a functional cytotoxic phenotype. Accordingly, mice bearing neo-epitope-specific CTLs also rejected a re-challenge with parental MC38 cell line (Figure 3h-k). These results support the notion that Trm cells are able to trigger the spreading of CTL responses against multiple highly relevant tumor-derived antigens with the potential to protect against tumors that are no longer recognized by Trm cells. These new data have been included as new Figure 3 and mentioned in the Results and Discussion sections.

- Figure 5: In this they only show the relationship between the different genetic signatures. This data is not enough to claim that the Trm have an effect on the maturation of DCs. At least this figure should be completed with a comparison with the transcriptomics analysis of the Trm cells the authors are working with.

We would like to stress that we are not claiming that Trm cells have an effect on DC maturation based on the RNA seq data alone. The purpose of this analysis was to examine whether the human data support our hypothesis, which it turned out to be the case. Unfortunately, making comparisons based on the mouse Trm cell gene signature in a human database is not very informative. Species-specific differences in gene signatures make correlations difficult to interpret. We have reworded the last paragraph of the Results section to make more clear that the analyses in the TCGA suggest that a similar Trm-DC crosstalk observed in mice may occur in human melanoma.

- Discussion: The final message of the discussion should indicate how the findings of the manuscript will have an impact on the immunotherapy.

We have incorporated a paragraph at the end of the Discussion section addressing this important topic.

Minor comments: 

- The scales of graphs in figure 1 and 2 should be normalized.

We have now normalized all graph scales in Figures 1 and 2.

**Reviewers Figure 1. Comparison between anti-CD8 antibody-depletion and FTY720 treatment.** C57BL/6 mice were intraperitoneally injected with tree 20µg doses of isotype-matched control (αCTRL) or anti-CD8 (αCD8) antibodies during three consecutive days or with seven 25µg doses of FTY720 during seven consecutive days. Analysis of CD8+ T cells in blood was performed 8 days after the beginning of each treatment. (a) Experimental scheme. (b) Representative dot-plots (left) and quantification as frequency of live cells (right) of CD8^+^ T cells in blood 8 days after the start of the treatments. Data corresponds to a representative experiment. Bars are the mean ± SEM, n=5 mice per group. **p<0.01 by Mann-Whitney unpaired t test.

[Figure Redacted]

**Reviewers Figure 3. B16F10 model with tamoxifen-induced OTI expression.** B16F10-iOTI cells, a model of B16F10 expressing the OTI epitope (OVA_257-264_) inducible by tamoxifen, were generated. B16F10-iOTIx5-ZsGreen cells (B16F10-iOTI) were generated by double retroviral transduction of B16F10 cell line with the pRetroQ-Cre-ERT2 vector and pMSCV-loxp-dsRed-loxo- OT1 × 5-ZsGreen vector encoding the OVA (257-264) (OTI) epitope minigene fused to ZsGreen. C57BL/6 CD45.1/1 mice were intradermally vaccinated with pVAX-OVA, 3 weeks later were intradermally challenged with B16F10-iOTI tumor. (a) Experimental scheme. (b) Graph showing the mean of tumor volume in vaccinated (blue) and unvaccinated control (black) groups. Data correspond to a representative experiment, n=7 and n=3 for vaccinated and controls groups respectively. Bars are the mean ± SEM. **** p ≤ 0.0001 by two-way ANOVA Bonferroni post-hoc test.

References:

1.	Galvez-Cancino, F., et al., *Vaccination-induced skin-resident memory CD8(+) T cells mediate strong protection against cutaneous melanoma.* Oncoimmunology, 2018. 7(7): p. e1442163.

2.	Matloubian, M., et al., *Lymphocyte egress from thymus and peripheral lymphoid organs is dependent on S1P receptor 1.* Nature, 2004. 427(6972): p. 355-60.

3.	Jiang, X., et al., *Skin infection generates non-migratory memory CD8+ T(RM) cells providing global skin immunity.* Nature, 2012. 483(7388): p. 227-31.

4.	Enamorado, M., et al., *Enhanced anti-tumour immunity requires the interplay between resident and circulating memory CD8+ T cells.* Nat Commun, 2017. 8: p. 16073.

5.	Li, M.H., T. Hla, and F. Ferrer, *FTY720 inhibits tumor growth and enhances the tumor-suppressive effect of topotecan in neuroblastoma by interfering with the sphingolipid signaling pathway.* Pediatr Blood Cancer, 2013. 60(9): p. 1418-23.

6.	Tsai, H.C., et al., *Effects of sphingosine-1-phosphate receptor 1 phosphorylation in response to FTY720 during neuroinflammation.* JCI Insight, 2016. 1(9): p. e86462.

7.	Ntranos, A., et al., *FTY720 impairs CD8 T-cell function independently of the sphingosine-1-phosphate pathway.* J Neuroimmunol, 2014. 270(1-2): p. 13-21.

8.	Azuma, H., et al., *Marked prevention of tumor growth and metastasis by a novel immunosuppressive agent, FTY720, in mouse breast cancer models.* Cancer Res, 2002. 62(5): p. 1410-9.

9.	Ng, K.T., et al., *Marked suppression of tumor growth by FTY720 in a rat liver tumor model: the significance of down-regulation of cell survival Akt pathway.* Int J Oncol, 2007. 30(2): p. 375-80.

10.	Thompson, E.D., et al., *Tumor masses support naive T cell infiltration, activation, and differentiation into effectors.* J Exp Med, 2010. 207(8): p. 1791-804.

11.	Mackay, L.K., et al., *The developmental pathway for CD103(+)CD8+ tissue-resident memory T cells of skin.* Nat Immunol, 2013. 14(12): p. 1294-301.

12.	Anderson, K.G., et al., *Intravascular staining for discrimination of vascular and tissue leukocytes.* Nat Protoc, 2014. 9(1): p. 209-22.

13.	Gubin, M.M., et al., *Checkpoint blockade cancer immunotherapy targets tumour-specific mutant antigens.* Nature, 2014. 515(7528): p. 577-81.

14.	Yadav, M., et al., *Predicting immunogenic tumour mutations by combining mass spectrometry and exome sequencing.* Nature, 2014. 515(7528): p. 572-6.

15.	Overwijk, W.W., et al., *Tumor regression and autoimmunity after reversal of a functionally tolerant state of self-reactive CD8+ T cells.* J Exp Med, 2003. 198(4): p. 569-80.

16.	Broz, M.L., et al., *Dissecting the tumor myeloid compartment reveals rare activating antigen-presenting cells critical for T cell immunity.* Cancer Cell, 2014. 26(5): p. 638-52.

17.	Clark, R.A., et al., *Skin effector memory T cells do not recirculate and provide immune protection in alemtuzumab-treated CTCL patients.* Sci Transl Med, 2012. 4(117): p. 117ra7.

18.	Malik, B.T., et al., *Resident memory T cells in the skin mediate durable immunity to melanoma.* Sci Immunol, 2017. 2(10).

19.	Enamorado, M., et al., *Enhanced anti-tumour immunity requires the interplay between resident and circulating memory CD8(+) T cells.* Nat Commun, 2017. 8: p. 16073.
